# Synthesis, Characterization, DNA/HSA Interactions, and Anticancer Activity of Two Novel Copper(II) Complexes with 4-Chloro-3-Nitrobenzoic Acid Ligand

**DOI:** 10.3390/molecules26134028

**Published:** 2021-07-01

**Authors:** Zhen-Fang Zeng, Qiu-Ping Huang, Jie-Hui Cai, Guang-Jin Zheng, Qiu-Chan Huang, Zi-Lu Liu, Zi-Lu Chen, You-Huan Wei

**Affiliations:** 1 School of Chemical and Biological Engineering, Guangxi Normal University for Nationalities, 23 Fozi Road, Chongzuo 532200, China; huangqiuping@gxnun.edu.cn (Q.-P.H.); caijiehui@gxnun.edu.cn (J.-H.C.); zhengguangjin0624@163.com (G.-J.Z.); huangqiuchan@163.com (Q.-C.H.); liuzilu0624@163.com (Z.-L.L.); 2 State Key Laboratory for the Chemistry and Molecular Engineering of Medicinal Resources, School of Chemistry and Pharmacy, Guangxi Normal University, 15 Yucai Road, Guilin 541004, China

**Keywords:** anticancer, apoptosis, copper(II) complex, cytotoxicity, DNA/HSA

## Abstract

The purpose of this study was to identify new metal-based anticancer drugs; to this end, we synthesized two new copper(II) complexes, namely [Cu(ncba)_4_(phen)] (**1**) and [Cu(ncba)_4_(bpy)] (**2**), comprised 4-chloro-3-nitrobenzoic acid as the main ligand. The single-crystal XRD approach was employed to determine the copper(II) complex structures. Binding between these complexes and calf thymus DNA (CT-DNA) and human serum albumin (HSA) was explored by electronic absorption, fluorescence spectroscopy, and viscometry. Both complexes intercalatively bound CT-DNA and statically and spontaneously quenched DNA/HSA fluorescence. A CCK-8 assay revealed that complex **1** and complex **2** had substantial antiproliferative influences against human cancer cell lines. Moreover, complex **1** had greater antitumor efficacy than the positive control cisplatin. Flow cytometry assessment of the cell cycle demonstrated that these complexes arrested the HepG2 cell cycle and caused the accumulation of G0/G1-phase cells. The mechanism of cell death was elucidated by flow cytometry-based apoptosis assays. Western blotting revealed that both copper(II) complexes induced apoptosis by regulating the expression of the Bcl-2(Bcl-2, B cell lymphoma 2) protein family.

## 1. Introduction

Carcinogenesis transforms normal cells into cancer cells in response to tissue injury or the influence of neighboring aging cells. The latter promote inflammation and tissue remodeling, and use secretome and paracrine regulation to reprogram differentiated cells into pluripotent cells associated with malignant growth [1]. In order to eliminate cancer cells, we must understand their characteristics and identify the factors that cause them to overproliferate. Cancers sustain proliferative signaling, resist cell death, deregulate cellular energetics, evade growth repressors, stimulate angiogenesis, activate infiltration and metastasis, enable replicative immortality, avoid immune destruction, support tumor-promoting inflammation, and cause genome instability and mutation [2]. The relationship between apoptosis and cancer forms the basis of innovative cancer therapy. 

Metal-based anticancer agents are among the most successful chemotherapeutic drugs. Cisplatin was the first metal-based drug administered to treat various solid tumors including testicular and ovarian cancers [3], bladder cancer and melanoma [4], and non-small cell lung cancers [5]. In clinical practice, however, the toxicity profile of cisplatin includes nausea and vomiting, renal dysfunction, neurotoxicity, and ototoxicity [6]. Analogs such as carboplatin and oxaliplatin are well tolerated in comparison to cisplatin but may cause innate, as well as acquired, resistance, neurotoxicity, nausea, and nephrotoxicity [7,8]. Hence, novel non-Pt (II/IV) anticancer complexes with fewer side effects are urgently required [9,10]. Copper(II) complexes have attracted attention in medicinal inorganic chemistry research [11,12,13,14,15,16]. Copper(II) is an essential micronutrient and a structural and catalytic cofactor in numerous biochemical pathways. It has demonstrated anticancer activity and could be a formulant in innovative anticancer drugs [17,18,19]. Copper(II) complexes, such as hydroxytryptophan copper(II) [20], substituted derivatives of pregnenolone acetate copper(II) [21], thiosemicarbazone copper(II) [22], substituted carboxylic acid copper(II) [23], 1,10-phenanthroline derivative copper(II) [24], pyridine derivative copper(II) [25], thiosemicarbazone copper(II) [26], piperazine copper(II) [27], and schiff copper(II) [28], have been synthesized, and their anticancer efficacy has been assessed.

Although substituted benzoic acids are often used as ligands to synthesize metal complexes [29,30], 4-chloro-3-nitrobenzoic acid copper(II) complexes have not been reported, in order to obtain new possible metal anticancer drugs, herein, we synthesized the copper(II) complexes [Cu(ncba)_4_(phen)] (**1**) and [Cu(ncba)_4_(bpy)] (**2**) and characterized them via carbon hydrogen nitrogen sulfur (CHNS) analysis, infrared spectroscopy, and single-crystal XRD. We investigated their interactions with CT-DNA/HSA and used a Cell Counting Kit 8 (CCK-8) assay to evaluate their cytotoxicity in vitro against human hepatocellular carcinoma (HepG2), human cervical carcinoma (HeLa), and human lung carcinoma (A549) cancer cell lines. We employed flow cytometry to explore HepG2 cell cycle distribution and induction of apoptosis. We also assayed caspase and Bcl-2 protein family expression in response to copper(II) complex exposure. Both complexes intercalatively bound CT-DNA and statically and spontaneously quenched DNA/HSA fluorescence. In vitro cytotoxicity assays demonstrated that complex **1** was more cytotoxic to HepG2, HeLa, and A549 cancer cells than complex **2** or cisplatin. Complex **1** and complex **2** arrested the HepG2 cell cycle and induced G0/G1.

## 2. Materials and Methods

### 2.1. Chemicals and Cell Lines

All reagents were used as purchased without any purification. Ethidium bromide (EB), CT-DNA, and HSA were supplied by Sigma-Aldrich Corp. (Shanghai, China). The human hepatocellular carcinoma HepG2, human cervical carcinoma HeLa, and the human lung carcinoma A549 cells were commercially provided by the Chinese Academy of Sciences Cell Bank/Stem Cell Bank and Council Cell Bank of Typical Culture Preservation (Shanghai, China). The other chemicals were provided by Sinopharm Chemical Reagent Co. Ltd. (Shanghai, China). All reagents were analytical grade. We prepared a stock CT-DNA solution in 5 mM Tris-HCl/50 mM NaCl buffer (pH 7.2) and determined its concentration by measuring absorbance at 260 nm using the UV-8000 spectrophotometer (Yuanxi Corp., Shanghai, China) with a molar absorption coefficient of 6600 M^−1^·cm^−1^ [31]. A stock solution of HSA was prepared in Tris-HCl/NaCl; to determine the concentration of HAS, the UV-8000 spectrophotometer (Yuanxi Corp., Shanghai, China) was used to measure absorbance at 280 nm, and a molar absorption coefficient of 35,700 M^−1^·cm^−1^ was used to calculate the concentration [32]. All solutions were kept at 4 °C and used within 4 d. Double-distilled H_2_O was the solvent in all experiments.

The infrared (IR) spectra of their ligands and of their respective copper(II) complexes were recorded within the limits of 4000–400 cm^−1^ using a Spectrum 65 FT/IR spectrometer (PerkinElmer, Waltham, MA, USA) with KBr pellets. Carbon, nitrogen, as well as hydrogen were quantitated in a Vario EL cube elemental analyzer (Elemantar, Langenselbold, Hesse, GER). The UV-8000 spectrophotometer (Yuanxi Corp., Shanghai, China) in the 200–1100 nm range was employed to read the electronic spectra. The fluorescence experiment was performed on the RF-5301PC spectrofluorometer (Shimadzu Corp., Kyoto, Japan) fitted with a 1.0 cm quartz cell. Viscosity was determined with the Ubbelohde viscometer (0.7–0.8-mm; Shenyi Glass Products Co. Ltd., Shanghai, China). In vitro cytotoxicity assays and Western blot analyses were conducted on a multifunctional microplate reader (Multiskan Spectrum, Thermo Fisher Scientific, Waltham, MA, USA). Cell cycle along with apoptosis assays were performed in a CyAn-ADP flow cytometry platform (Beckman Coulter, Brea, CA, USA).

### 2.2. Preparation

**Scheme 1:**
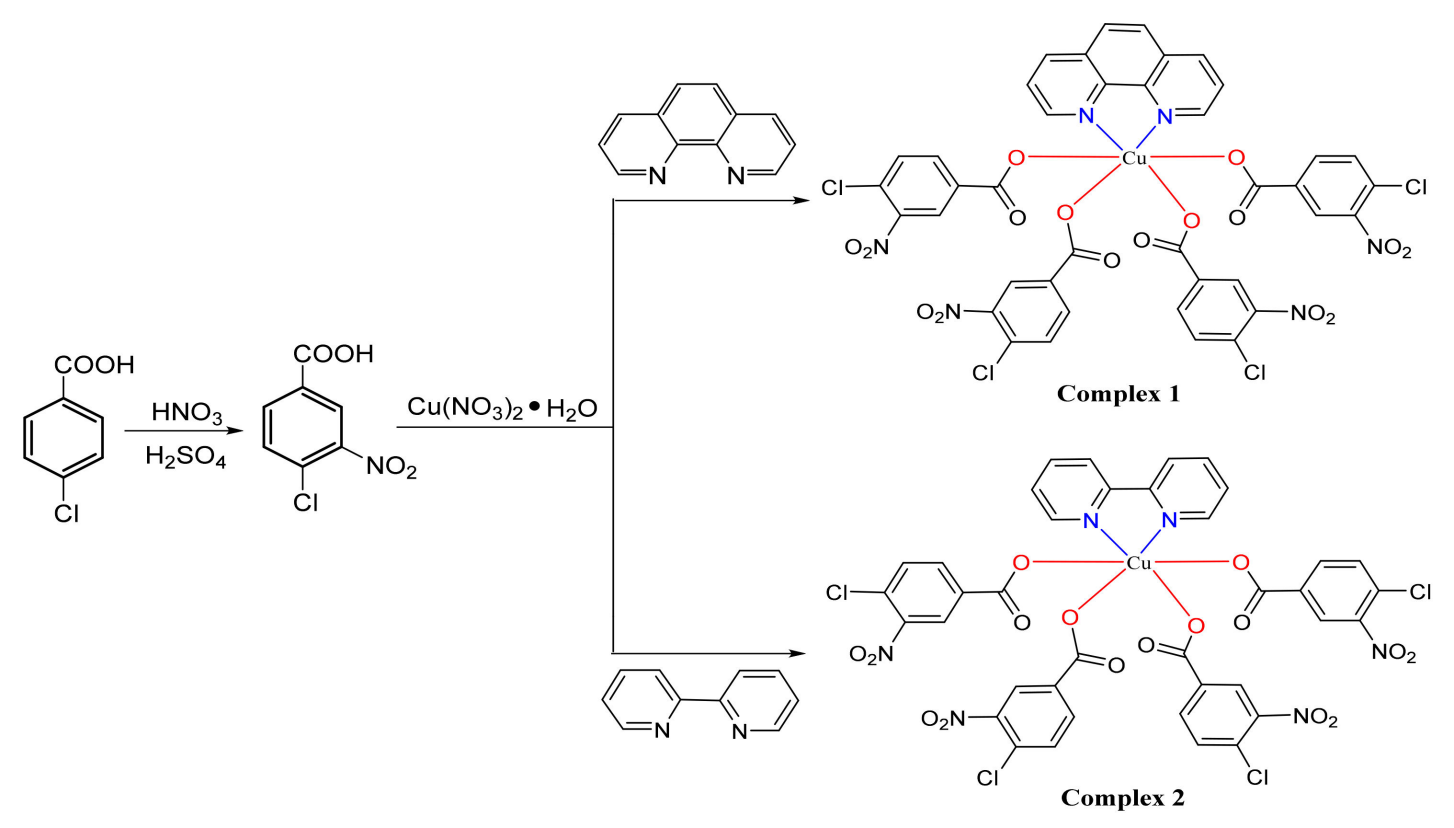
Synthetic routes of the ligand and complex **1** and complex **2**.

Complex **1** and complex **2** were prepared at high yield as depicted in Scheme 1.

#### 2.2.1. 4-Chloro-3-Nitrobenzoic Acid (ncba) Synthesis

To a 100 mL three-port flask, 25.0 mL of 98% (*w*/*v*) concentrated H_2_SO_4_ was added and the temperature was set to <15 °C. Then, 0.1 mol *p*-chlorobenzoic acid was slowly added with constant stirring. After the solid dissolved, the temperature was set to <15 °C, 20.0 mL of 68% (*w*/*v*) nitric acid was introduced, and the temperature was increased to 50–65 °C and maintained for five hours. Thereafter, the mixture was allowed to cool to 20–25 °C, poured into ice H_2_O, stirred, precipitated, filtered, and rinsed using water to obtain the unrefined product. The latter was recrystallized with ethanol and dried to obtain the finished product with the following physicochemical characteristics: melting point range, 180.0–182.0 °C; ^1^H NMR (400 MHz, D_2_O): δ 8.43 (s, 1H), 8.06 (d, *J* = 8.1 Hz, 1H), and 7.67 (d, *J* = 8.5 Hz, 1H).

#### 2.2.2. [Cu(ncba)_4_(phen)] (Complex **1**) Synthesis

A mixture of 0.45 mmol ncba (0.091 g) and 0.027 g (0.15 mmol) 1,10-phenanthroline (phen) was dissolved in absolute methanol (10.0 mL) and a methanolic solution of copper(II) nitrate trihydrate (0.024 g [0.10 mmol] in 15.0 mL) was introduced dropwise to the mixture. We stirred the solution for 30 min and left it to evaporate in a beaker at 20–25 °C. After several days, we collected well-formed blue crystals suitable for XRD and rinsed them with cold methanol. The final product (C_40_H_20_Cl_4_CuN_6_O_16_) yield was 63.4%, and its molecular weight was 1045.96. Its calculated and measured C, H, and N values were 45.93%, 1.93%, and 8.03% and 46.15%, 2.18%, and 8.12%, respectively. Its infrared (IR) (KBr, cm^−1^) data were v(=CH) 3438, v(C=O) 1695, v(C=C) 1541 and 1358, v(C=N) 1282, and v(C-N-C) 1047 and 776.

#### 2.2.3. [Cu(ncba)_4_(bpy)] (Complex **2**) Synthesis

Synthesis of complex **2** was performed in the same manner as complex **1** except 0.023 g (0.15 mol) 2,2′-bipyridine (bpy) was used instead of 1,10-phenanthroline. The final product (C_38_H_20_Cl_4_CuN_6_O_16_) yield was 52.5%, and its molecular weight was 1021.94. Its calculated and measured C, H, and N values were 44.66%, 1.97%, and 8.22% and 44.64%, 2.32%, and 8.12%, respectively. Its IR (KBr, cm^−1^) data were v(=CH) 3426 and 2995, v(C=O) 1597, v(C=C) 1455, v(C=N) 1316, and v(C-N-C) 1115, 758 and 621.

### 2.3. X-ray Structure Determination

Appropriate individual crystals of complex **1** and complex **2** were selected and mounted onto thin glass fibers. We collected the data at 296(2) K in a Bruker Smart ApexII CCD instrument (Bruker Corp., Karlsruhe, Germany) fitted with graphite monochromatic Mo-Ko radiation (λ = 0.71073 Å). The absorption effects were semi-empirically calibrated. Olex2 [33] was employed to solve the structures and refined with SHELXL-97 crystallographic software [34]. Non-hydrogen atoms were anisotropically refined. Hydrogen atoms bound to carbons were placed in geometrical idealized positions, followed by refinement with a riding model. All other hydrogens were localized in the final difference Fourier map. The full-matrix least-squares refinement final cycle was based on the observed reflections along with variable parameters. Crystallographic as well as structural refinement data for complex **1** and complex **2** are listed in Table 1. Selected bond lengths (Å) as well as angles (°) are listed in Table S1. CCDC 2013056 and CCDC 2013057 for complex **1** and complex **2** harbor supplementary crystallographic data were provided freely by the Cambridge Crystallographic Data Centre (Cambridge, UK).

### 2.4. DNA Binding Studies

Electronic absorption titration was employed to explore binding of DNA with metal complexes. We carried out the absorption titration tests via introducing 80 μL of 2 mM CT-DNA to 2.5 mL of 50 μM complex **1** and 3.0 mL of 50 μM complex **2**. The solutions were kept at 20 °C for 5 min to ensure complete reaction. The UV-8000 spectrophotometer (Yuanxi Corp., Shanghai, China) was used to measure the absorption spectra at 200–450 nm.

Fluorescence was measured using the RF-5301PC spectrofluorometer (Shimadzu Corp., Kyoto, Japan) fitted with a 1 cm quartz cuvette. Fluorescence spectra of both complexes were measured in the 500–700 nm range. The excitation wavelength was 565 nm for complex **1** and 491 nm for complex **2**. The excitation/emission slits were set to 5 nm. We conducted the scanning at 240 nm/min at a constant 4.0 μM EB and a constant 5.0 μM CT-DNA. Three milliliters buffer solution was used for complex **1**, and 2.5 mL buffer solution was used for complex **2**. The quantities of complex **1** or complex **2** (20 μL; 1 mM) were increased several folds. The complex-DNA solutions were left to stand 5 min before each measurement.

CT-DNA viscosity was measured in the Ubbelohde viscometer (0.7–0.8-mm; Shenyi Glass Products Co. Ltd., Shanghai, China) with and without complex **1** or complex **2** or EB. The samples were maintained at 29.0 ± 0.1 °C, and a digital electronic stopwatch was employed to measure the flow time. Viscosity was calculated based on the DNA-containing solution flow times corrected for buffer flow time (*t*_0_), *η* = (*t* − *t*_0_)/*t*_0_. Data were reported as (*η*/*η*_0_)^1/3^ relative to (*C*_complex_/*C*_DNA_), where *η* is the DNA viscosity in the presence of the complex, *η*_0_ is the DNA viscosity, *C*_complex_ is the quantity of the complex, and *C*_DNA_ is the DNA quantity. The latter was maintained at a constant 200 μM, and the complex quantities were varied to create [complex]/[DNA] ratios in the 0.00–0.30 range. The average time was computed from triplicate assessments.

### 2.5. HSA Binding

Absorption titration was performed by adding 5.0 μL of 0.30 μM complex **1** to 3.0 mL of 200 μM HSA and by adding 50 μL of 1.2 mM complex **2** to 2.5 mL of 200 μM HSA. The solutions were left to stand at 20 °C for 5 min for complete reaction. The UV-8000 spectrophotometer (Yuanxi Corp., Shanghai, China) was employed to record the absorption in the 200–400 nm range.

Fluorescence spectra were measured from 250–500 nm at an excitation wavelength of 280 nm. Both the excitation and emission slits were set to 10 nm. Scanning was conducted at 240 nm/min and a constant 5.0 μM HSA quantity. An amount of 3 mL of buffer solution was used for complex **1**, and 2.5 mL buffer solution was used for complex **2**. The quantities of complex **1** (10 μL, 1.2 mM), along with complex **2** (2 μL, 1.2 mM) were increased several folds. Complex–HSA solutions were left to stand for 5 min before each measurement.

### 2.6. In Vitro Cytotoxicity Assays

The cytotoxicity levels of complex **1** and complex **2** against HepG2, HeLa, and A549 cells were determined with a CCK-8 kit (Biosharp, Beijing, China) according to the manufacturer’s instructions. Briefly, log-phase HepG2, HeLa, and A549 cells were harvested, dissociated, and inoculated in triplicate on 96-well plates at 5 × 10^3^/well. The cells were then allowed to grow overnight at 37 °C under a 5% CO_2_/95% O_2_ atmosphere. Cells with or without the copper(II) complex were allowed to grow for 24 h, 48 h, 72 h, or 96 h and then inoculated in 10 μL CCK-8 + 90 μL medium in 10% (*v*/*v*) FBS in the dark for 3 h. Absorbance was read at 450 nm in a Multiskan Spectrum multifunctional microplate reader (Thermo Fisher Scientific, Waltham, MA, USA).

### 2.7. Cell Cycle Assays

Anticancer agents interfere with various cell cycle phases. The cancer cell cycle may help elucidate the DNA-binding copper(II) complex mechanisms. HepG2 cells were inoculated onto six-well plates at 1 × 10^5^/well at 37 °C overnight under a 5% CO_2_/95% O_2_ conditions. The cells were left untreated or subjected to complex **1** or complex **2** for 24 h, trypsinized, rinsed with cold PBS (phosphate-buffered saline, Hyclone Laboratories Inc., Logan, UT, USA), fixed using 70% (*v*/*v*) ethanol, and kept at 4 °C. Afterwards, the pellets were washed twice with 1.0 mL PBS. Then, 10.0 mL of 20 μg/mL RNase (Sigma-Aldrich Corp., Shanghai, China) and 10 μL of 50 μg/mL PI (propidium iodide, Sigma-Aldrich Corp., Shanghai, China) were introduced, and the cell suspensions allowed to grow for 0.5 h at 37 °C. The samples were then analyzed with a Beckman CyAn-ADP flow cytometry platform (Beckman Coulter, Brea, CA, USA). Ten thousand cells were analyzed per sample. A similar procedure was used to assay the HepG2 cell cycles after 48 h treatment with complex **1** or complex **2**.

### 2.8. Cell Apoptosis Assay

Cell culture was performed as described for the cell cycle assay. The cells were left untreated or subjected to complex **1** or complex **2** for 24 h, and then harvested, followed by rinsing twice with 1.0 mL PBS. One hundred microliters of binding buffer, 5 μL Annexin V-FITC along with 5 μL PI were introduced to the cell suspensions, and the latter were incubated at 25 °C in the dark for 15 min. Fluorescence was measured at 488 nm excitation in a Beckman CyAn-ADP flow cytometry platform (Beckman Coulter, Brea, CA, USA). Ten thousand cells were analyzed per sample. A similar procedure was used to assay HepG2 cell apoptosis after 48 h treatment with complex **1** or complex **2**.

### 2.9. Western Blot

HepG2 cells were lysed, and their protein contents were purified with RIPA lysis buffer (Beyotime, Shanghai, China) in 1% (*w*/*v*) phenylmethanesulfonyl fluoride (PMSF; Sigma-Aldrich Corp., Shanghai, China). Equivalent amounts of protein from each extract were fractionated on an SDS-PAGE gel and transfer-embedded onto PVDF membranes (EMD Millipore, Billerica, MA, USA). The membranes were then inoculated overnight with primary antibodies at 4 °C and rinsed thrice with Tris-buffered saline-Tween (TBST). The primary antibodies consisted of anti-caspase-3 (Abcam, Cambridge, UK; 1:1000), anti-Bcl-2 (Cell Signaling Technology, Danvers, MA, USA; 1:1000), anti-Bax (Cell Signaling Technology, Danvers, MA, USA; 1:1000), and anti-GAPDH (Cell Signaling Technology, Danvers, MA, USA; 1:5000) diluted in TBST. Goat immunoglobulin G (IgG) labeled with HRP (MultiSciences, Hangzhou, China; 1:2000) was introduced, and the membranes were incubated at 20 °C for 1 h. ECL detection reagent (Thermo Fisher Scientific, Waltham, MA, USA) was applied to detect the signals.

## 3. Results and Discussion

### 3.1. Synthesis and Characterization

**Figure 1:**
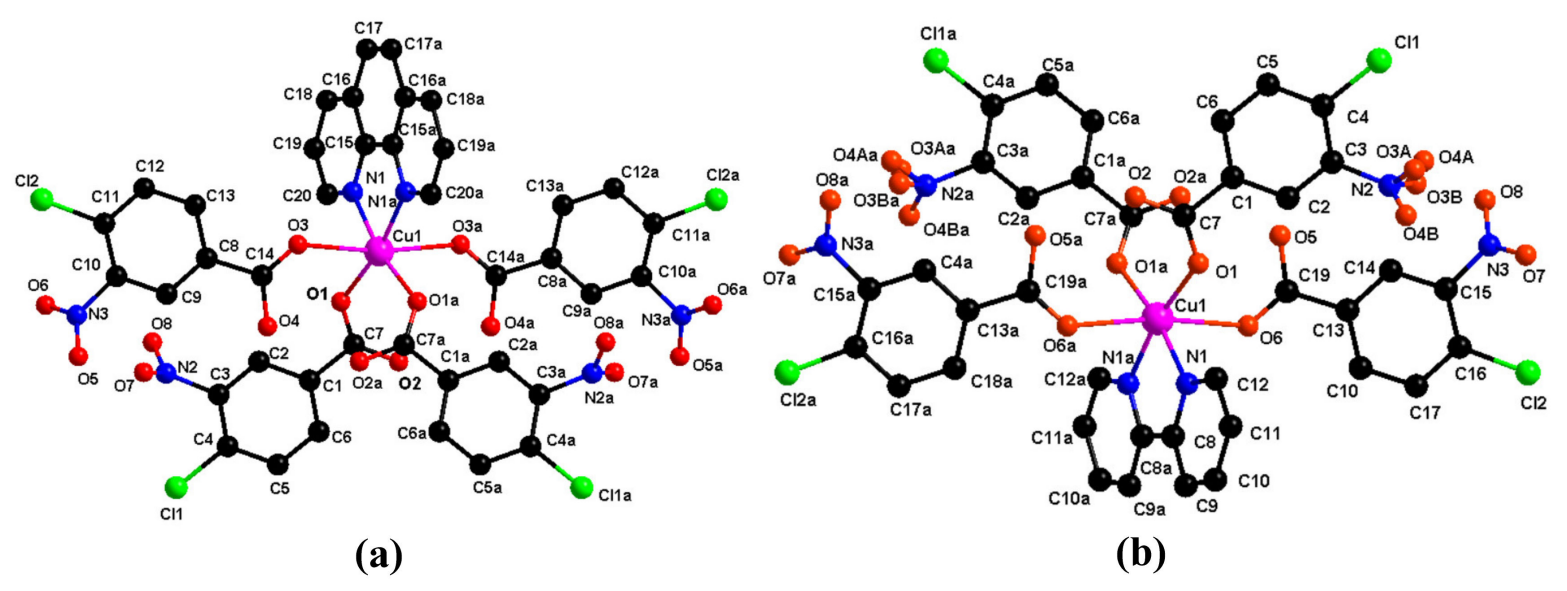
Crystalline structures of Cu(II) complex **1** (**a**) and Cu(II) complex **2** (**b**).

Here, we prepared two new copper(II) complexes using 4-chloro-3-nitrobenzoic acid ligand. Both are stable in air and soluble in dimethylformamide (DMF). Single-crystal X-ray analysis illustrated that complex **1** and complex **2** crystallized in the monoclinic crystal system with the C2/c space group (Figure 1). The Cu(II) ions in both complexes were hexacoordinated by four oxygen (O) atoms from the 4-chloro-3-nitrobenzoic acid ligand for complex **1** and by two nitrogen (N) atoms from phen and 2,2’-bpy for complex **2**. Table S1 shows that the Cu(II)-O and Cu(II)-N distances were in the ranges of 1.9354(17)–1.9380(18) Å and 1.999(2)–2.010(2) Å, respectively. The O/N-Cu-O/N bond angles were in the ranges of 81.90(11)–168.87(7)° for complex **1** and 81.25(13)–169.97(8)° for complex **2**.

### 3.2. DNA Binding Experiments

#### 3.2.1. Electronic Absorption

**Figure 2:**
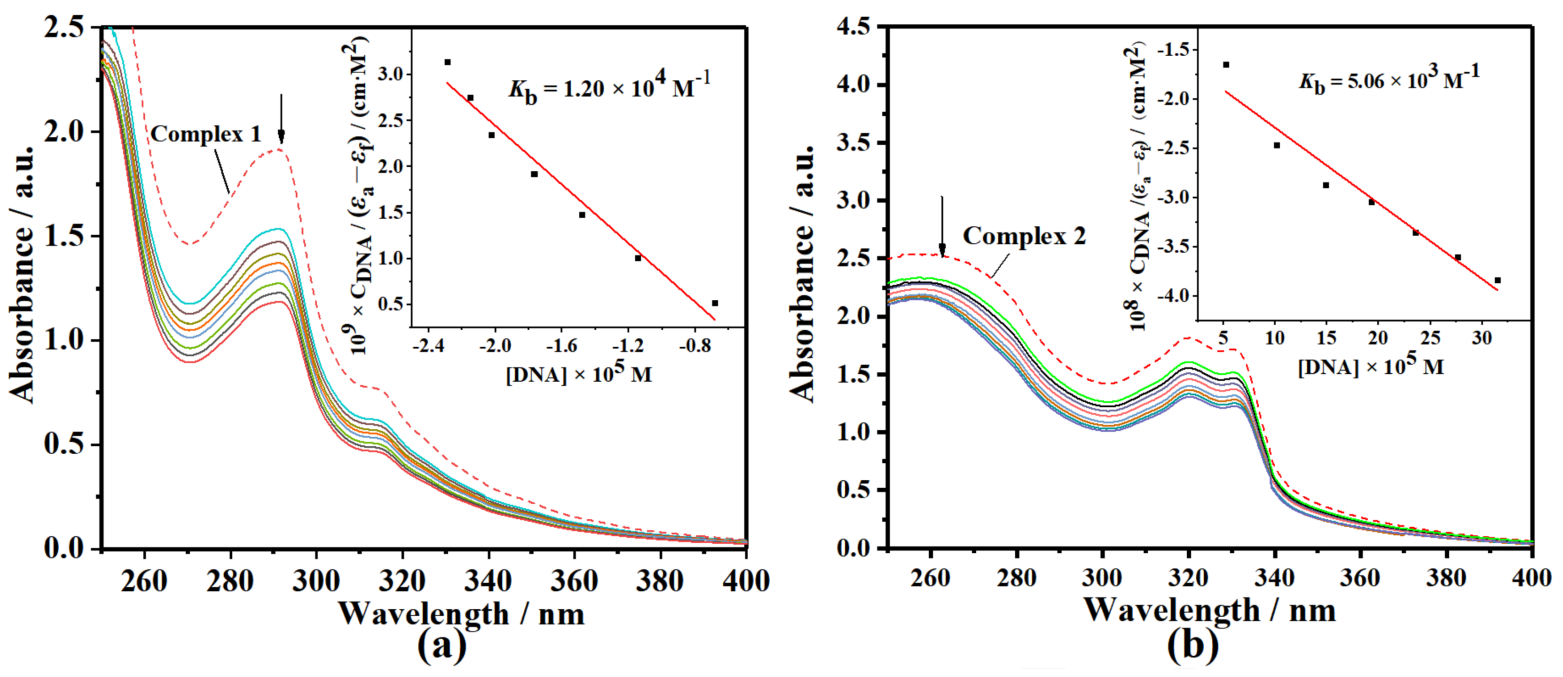
The electronic absorption spectra of complex **1** (**a**) and complex **2** (**b**) with or without CT-DNA. Arrows indicate changes in absorbance with escalating DNA quantity. Inset: [DNA]/(*ε*_a_ − *ε*_f_) vs. DNA] plots.

Electronic absorption spectroscopy reveals the binding characteristics of the metal complexes with DNA [35]. Intercalative binding of a complex to DNA causes hypochromism and redshift due to the remarkable stacking intercalation of the aromatic chromophore of the ligand with the base pairs of DNA. The magnitude of the hypochromism is proportional to the intercalative binding strength [36]. Figure 2 indicates the absorption spectra of the complexes with and without CT-DNA. The absorption band intensities exhibited negligible redshift, 23.90% hypochromism for complex **2**, and 35.83% hypochromism for complex **1**, in response to increasing DNA quantity. Hence, intercalative binding occurred between the DNA and each complex. The intrinsic binding constant (*K*_b_) was computed as shown in Equation (1) below [37]:

(1) $$\left[\mathrm{DNA}\right]/\left(\varepsilon_{a}-\varepsilon_{f}\right)=\left[\mathrm{DNA}\right]/\left[\left(\varepsilon_{b}-\varepsilon_{f}\right)+1/K_{b}\left(\varepsilon_{b}-\varepsilon_{f}\right)\right]$$

where *ε*_a_ designates the apparent complex extinction coefficient, *ε*_f_ designates free complex extinction coefficient, *ε*_b_ designates the bound complex extinction coefficient, and [DNA] designates the CT-DNA quantity. In the [DNA]/(*ε*_a_ − *ε*_f_) vs. [DNA] plot, *K*_b_ is the slope: intercept ratio. The calculated binding constants were 1.20 × 10^4^ M^−1^ and 5.06 × 10^3^ M^−1^ for complex **1** and complex **2**, respectively. Complex **1** had larger binding constants and greater hypochromism than complex **2**. The calculated intrinsic equilibrium binding constants (*K*_b_) (10^3^–10^5^ M^−1^) were consistent with literature values [38]. Hence, there was strong affinity between the complexes and the DNA.

#### 3.2.2. Fluorescence Spectroscopy

EB complexes with DNA and strongly fluoresces because of the interaction of the planar phenanthridine rings with the neighboring DNA base pairs. A competitive reaction takes place when the complex co-exists with EB-DNA. The EB complex dissociates from the DNA double helix and decreases fluorescence. Therefore, EB is a fluorescent probe for the DNA [39].

**Figure 3:**
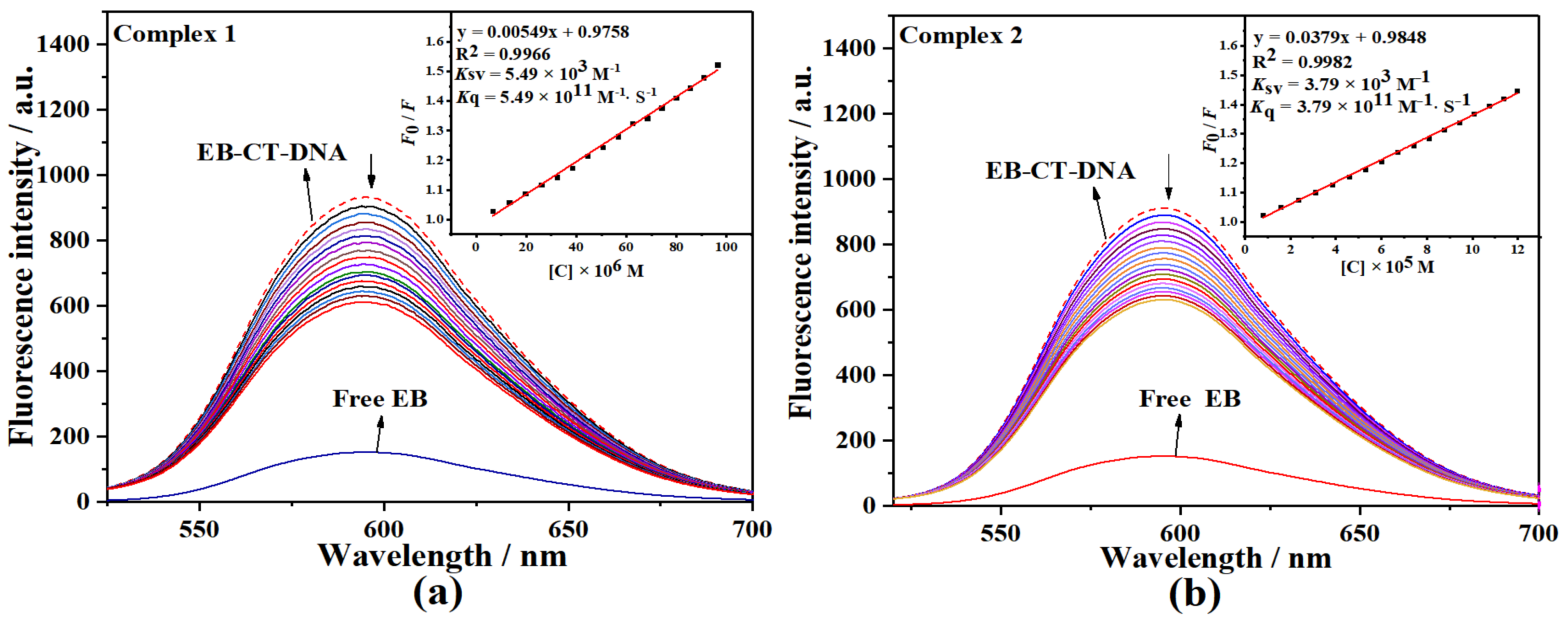
EB-DNA emission spectra in the absence or the presence of complex **1** (**a**) and complex **2** (**b**). Inset: *F*_0_/*F* vs. [C] plots. Arrows indicate the changes in intensity of fluorescence with escalating complex quantity.

**Figure 4:**
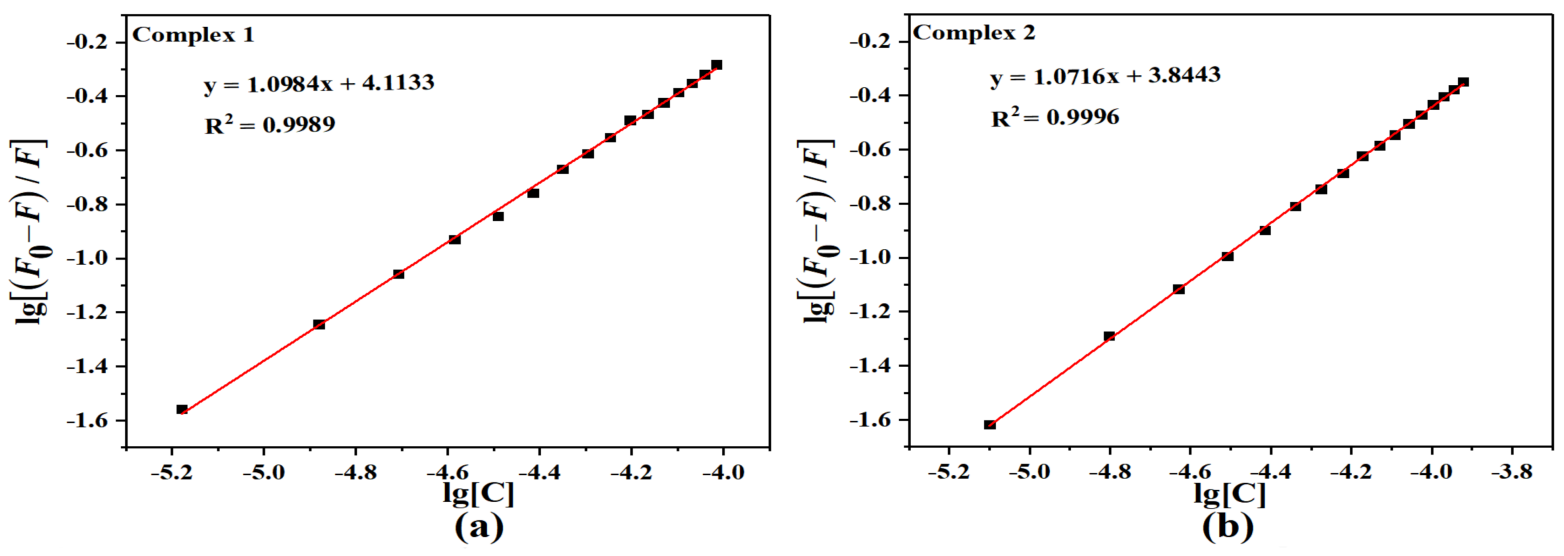
Double-log plots of DNA fluorescence quenching by complex **1** (**a**) and complex **2** (**b**).

Figure 3 illustrates the alterations in the fluorescence emission spectra of the complexes with CT-DNA. Emission intensity decreased in response to the addition of complex **1**, as well as complex **2**. The magnitude of quenching resembled that of externally binding molecules [40]. Complex fluorescence quenching by CT-DNA is quantitated by the classical Stern–Volmer Equation (2) [41]:

(2) $$F_{0}/F=1+K_{q}\tau_{0}\left[C\right]=1+K_{\mathrm{sv}}\left[C\right]$$

where *F*_0_ designates the intensity of fluorescence without the quencher, and *F* designates the intensity of fluorescence with the quencher: *τ*_0_ (~10^−8^ s) is the fluorophore lifespan, [C] is the quencher quantity, *K*_sv_ designates the Stern–Volmer quenching, and *K*_q_ designates the quenching rate constant. The *F*_0_/*F* vs. [C] plot is linear, and *K*_sv_ is calculated from its slope. The quenching constants (*K*_sv_) for complex **1** and complex **2** were 5.49 × 10^3^ M^−1^ and 3.79 × 10^3^ M^−1^, respectively. *K*_q1_ = 5.49 × 10^11^ M^−1^·s^−1^ and *K*_q2_ = 3.79 × 10^11^ M^−1^·s^−1^. Therefore, quenching efficiency was high [42]. The foregoing *K*_q_ were markedly higher relative to the biomolecule limiting diffusion constant *K*_dif_ (2.0 × 10^l0^ M^−1^·s^−1^) [43]. For this reason, the fluorescence quenching was probably static [44]. In that case, the relationship of the intensity of fluorescence with [C] is described by Equation (3) [45,46]:

(3) $$\log\left[\left(F_{0}-F\right)/F\right]=\log K_{a}+n\log\left[C\right]$$

where *n* designates the number of binding sites, and *K*_a_ designates the binding constant. Figure 4 shows that the *K*_a_ were acquired from the intercepts and slopes of the log[(*F*_0_ − *F*)/*F*] vs. log[C] plots. *K*_a_ = 1.30 × 10^4^ M^−1^ and 6.99×10^3^ M^−1^ while *n* = 1.0984 and 1.0716, respectively, for complex **1** and complex **2**.

#### 3.2.3. Viscosity Measurements

**Figure 5:**
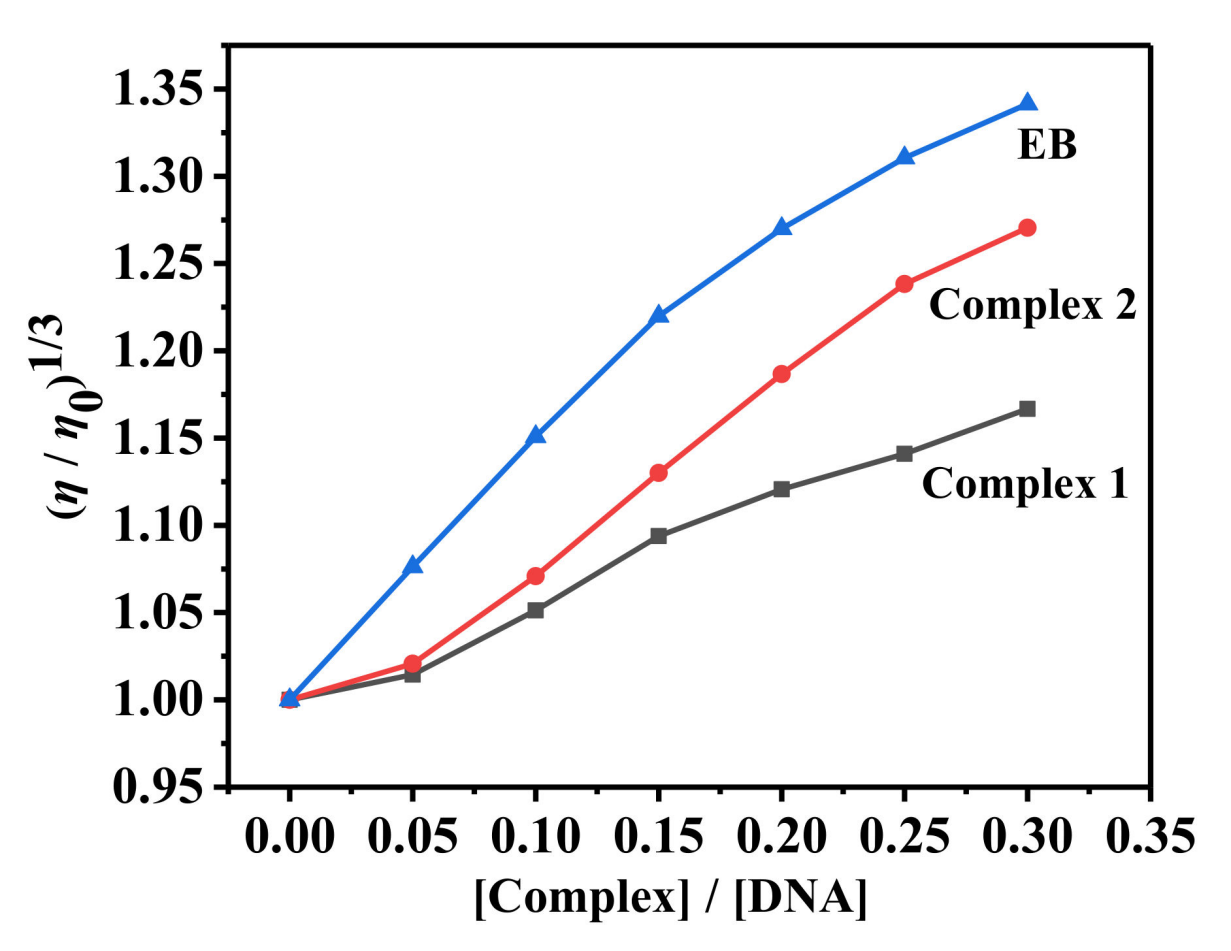
Impacts of increasing complex **1**/complex **2** numbers, as well as EB on relative DNA viscosity at 29.0 ± 0.1 °C.

We evaluated the changes DNA solution viscosity upon the addition of the complexes and the EB to clarify the interactions between CT-DNA and the complexes. The viscosity was measured to assess the complex–DNA binding mode because DNA viscosity increases with DNA length [47]. Intercalation elongates the DNA helices and increases DNA viscosity. The base pairs are disassociated to accommodate the binding ligands. On the contrary, non-classical or partial intercalation bends the DNA helices and lowers DNA viscosity. Neither grooving nor electrostatic binding affects DNA viscosity [48]. Figure 5 shows that DNA viscosity increases with the [complex]/[DNA] ratio. Therefore, EB and both complexes bind intercalatively to DNA, and this finding was corroborated by the absorption and emission spectra.

### 3.3. HSA Binding Experiments

**Figure 6:**
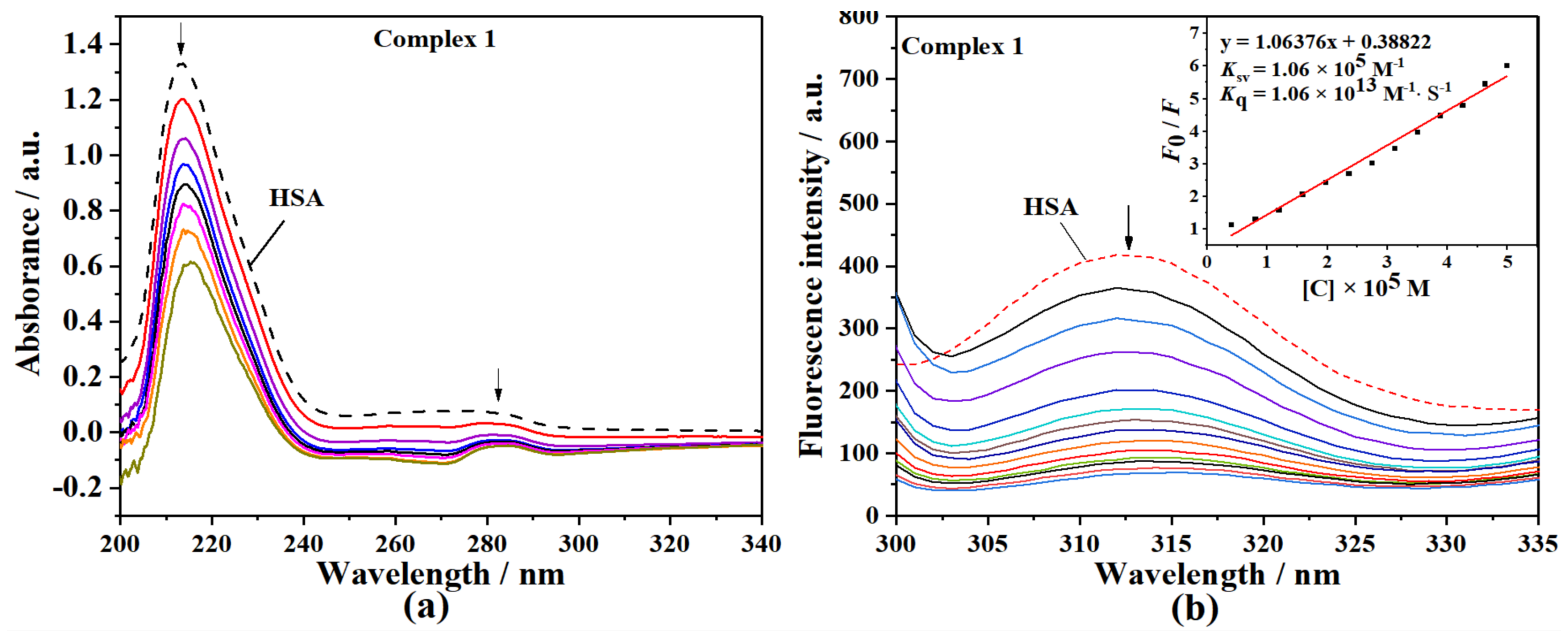
HSA electronic absorption spectra with and without complex **1** (**a**). HSA platform emission spectra with and without complex **1** (**b**). Inset: *F*_0_/*F* vs. [C] plot. Arrows illustrate the intensity changes with increasing complex quantity.

**Figure 7:**
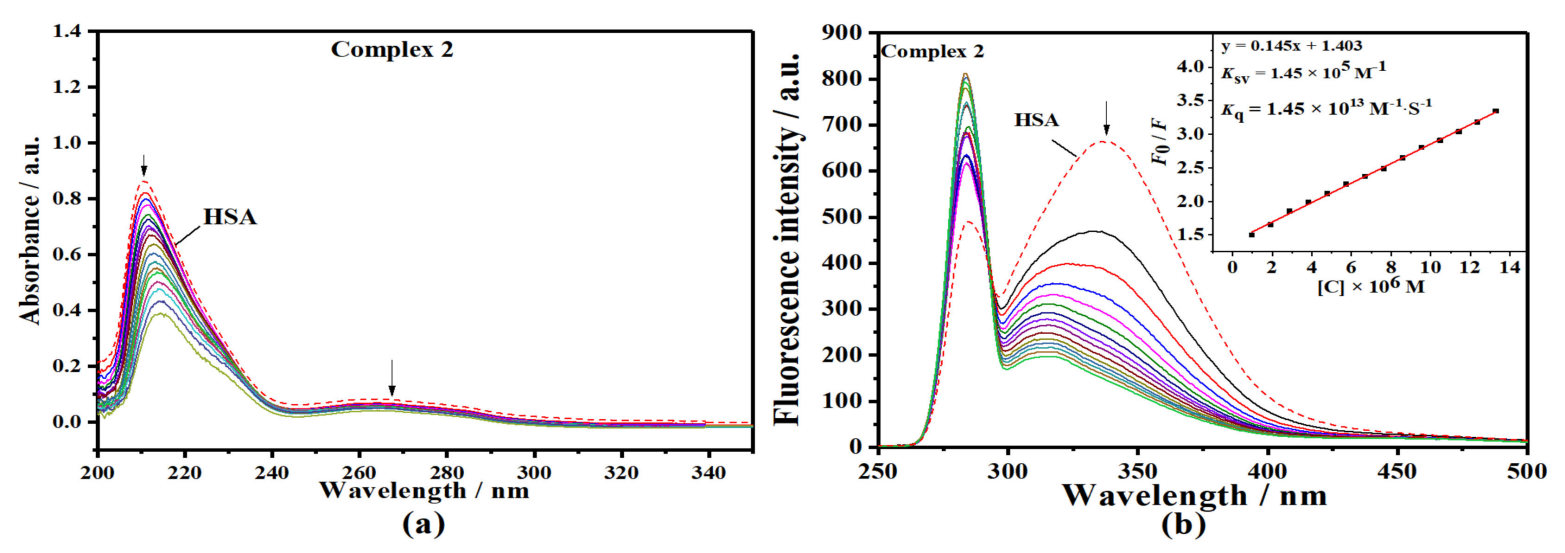
HSA electronic absorption spectra with and without complex **2** (**a**). HSA platform emission spectra with and without complex **2** (**b**). Inset: *F*_0_/*F* vs. [C] plot. Arrows illustrate the changes in intensity with increasing quantity of the complex.

The procedure used to study the interactions between HSA and the complexes was the same as that used to evaluate the interactions between DNA and the complexes. In the former case, however, no fluorophore (EB) was required. The absorption bond intensities became hypochromic with increasing complex **1** and complex **2** quantity. Figure 6 and Figure 7 show that the quenching constants (*K*_sv_) were 1.06 × 10^5^ for complex **1**, as well as 1.45 × 10^5^ M^−1^ for complex **2**. *K*_q1_ = 1.06 × 10^13^ M^−1^·s^−1^ and *K*_q2_ = 1.45 × 10^13^ M^−1^·s^−1^. *K*_a_ were 6.63 × 10^6^ and 1.84 × 10^3^ M^−1^ while *n* were 1.42 and 0.60 for complex **1** and for complex **2**.

### 3.4. Cytotoxicity Assay In Vitro

**Figure 8:**
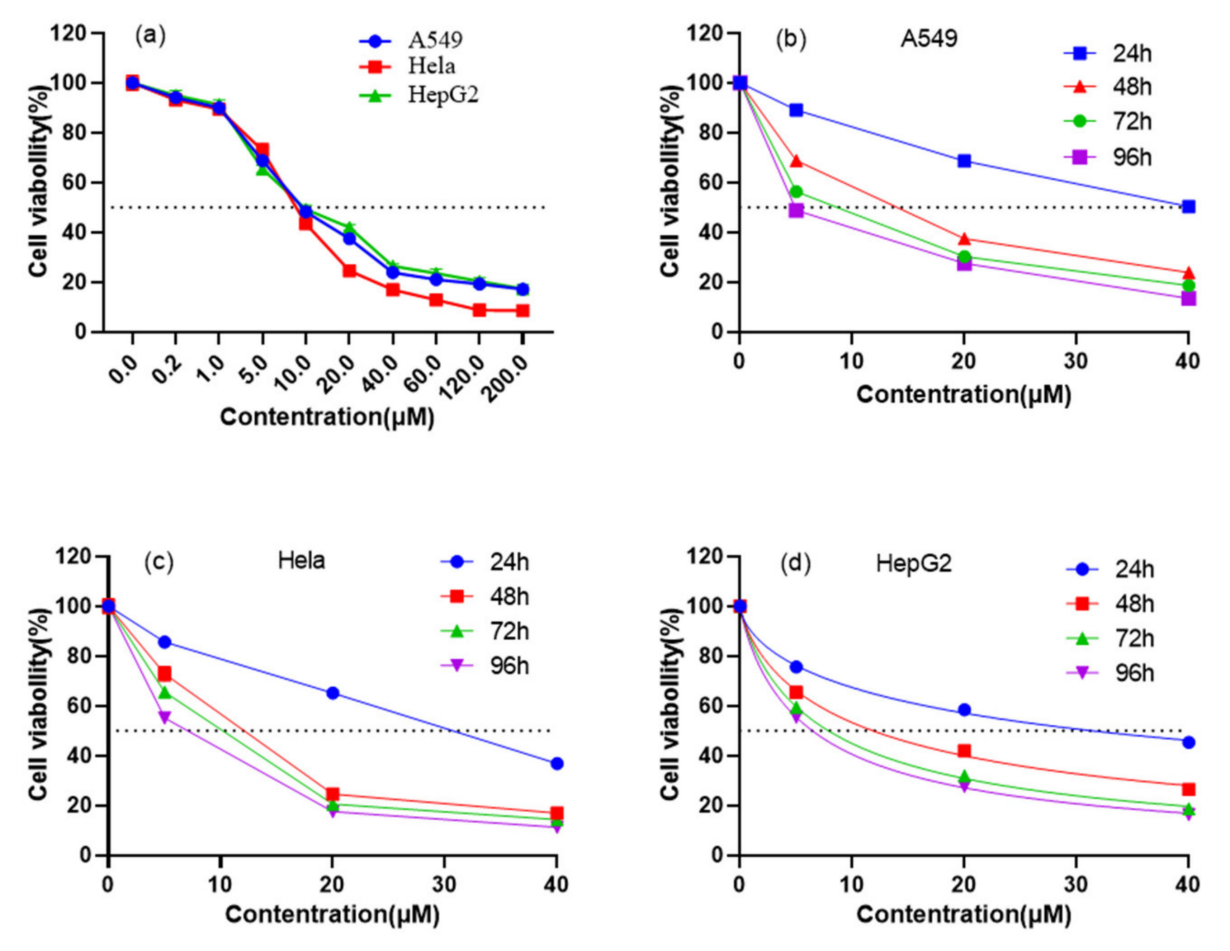
(**a**) Effects of complex **1** on HepG2, HeLa, and A549 cell viability and proliferation after 48 h exposure. (**b**–**d**) Time course of effects of complex **1** on A549, HeLa, and HepG2 cell viability and proliferation.

**Figure 9:**
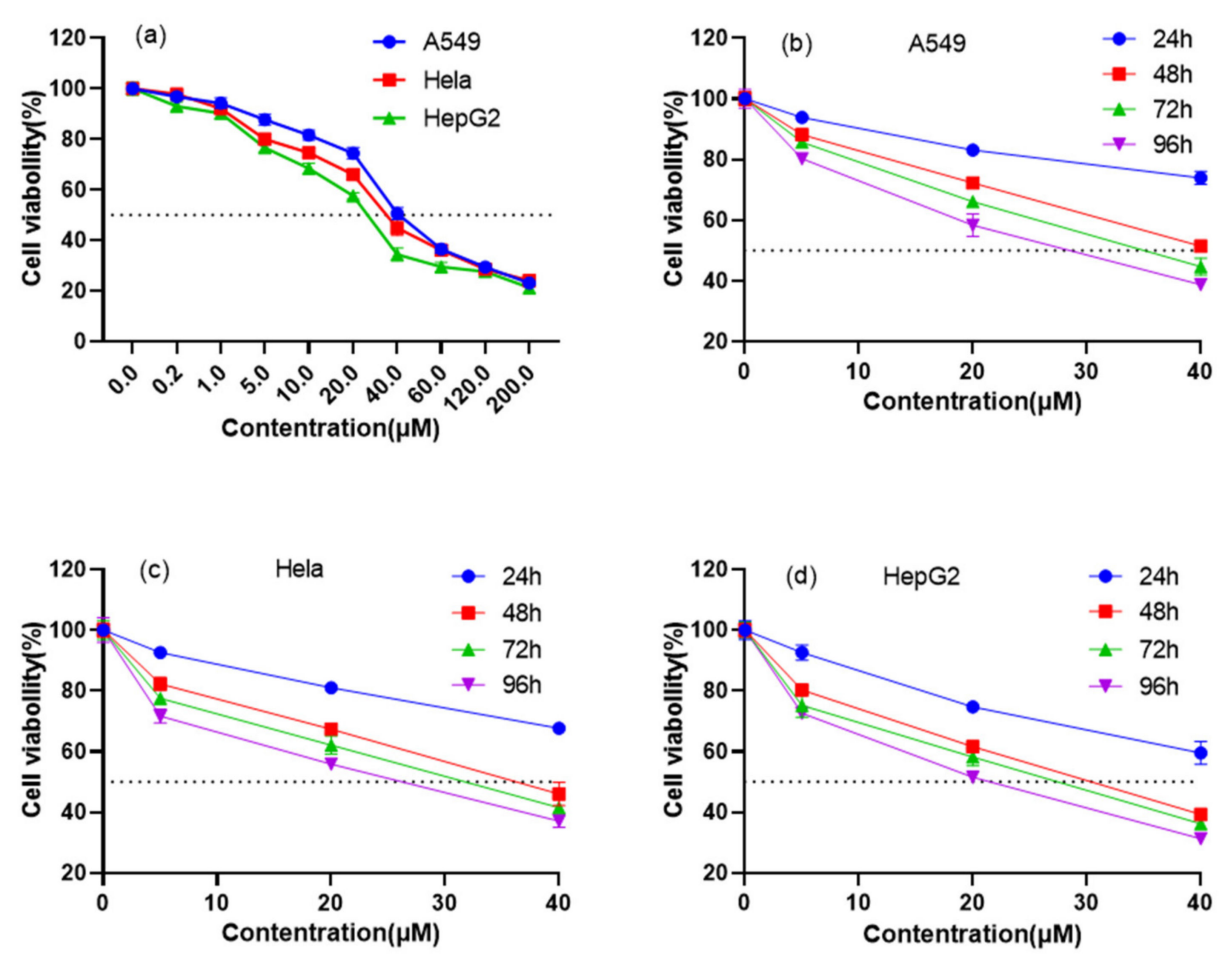
(**a**) Effects of complex **2** on HepG2, HeLa, and A549 cell viability and proliferation after 48 h exposure. (**b**–**d**) Time course of effects of complex **2** on A549, HeLa, and HepG2 cell viability and proliferation.

Complex cytotoxicity was tested on A549, HeLa, and HepG2 cell lines by the CCK-8 cell survival assay. A549, HeLa, and HepG2 were subjected to various complex **1** and complex **2** quantities for 24 h, 48 h, 72 h, as well as 96 h. Complex **1** had IC_50_ = 8.99 μM, 11.32 μM, and 12.19 μM against HeLa, A549, and HepG2 cells, respectively, after 48 h (Figure 8a). Figure 9a shows that complex **2** had IC_50_ = 24.46 μM, 35.92 μM, and 44.85 μM against HepG2, HeLa, and A549 cells, respectively. The IC_50_ of complex **1** and complex **2** against A549, HeLa, as well as HepG2 cells at various time points are listed in Table 2. Both complexes displayed strong antiproliferative influence against A549, HeLa, as well as HepG2 cells. However, complex **1** had stronger activity against all three cell lines than cisplatin and exhibited the highest activity against HeLa cells at 48 h. Cell viability was both dose- and time-dependent. Hence, complex **1** and complex **2** gradually penetrated the cells, inducing cell death.

### 3.5. Cell Cycle Arrest

**Figure 10:**
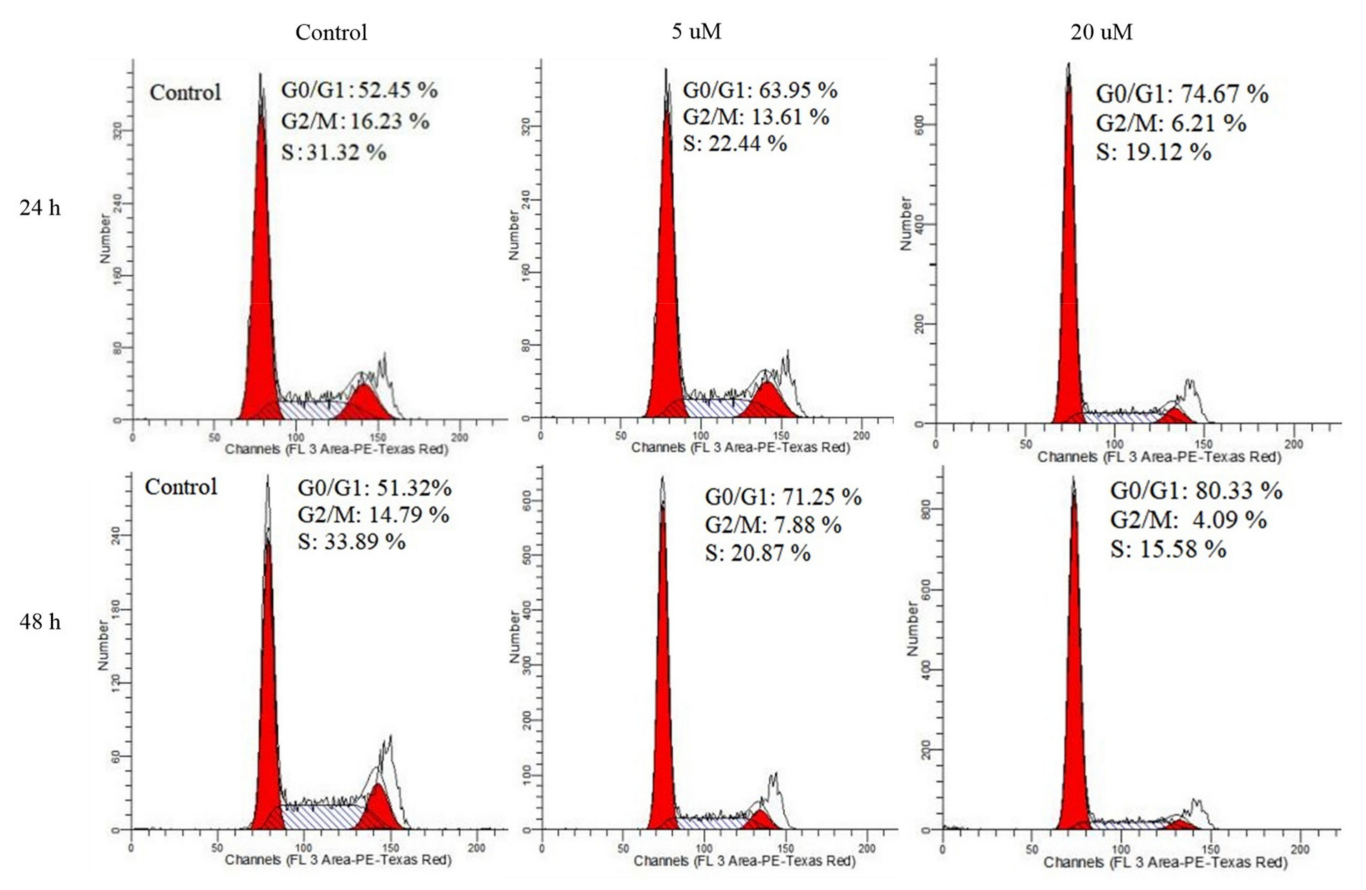
HepG2 cell cycle distributions after exposure to 5 μM, as well as 20 μM complex **1** for 24 h and 48 h.

**Figure 11:**
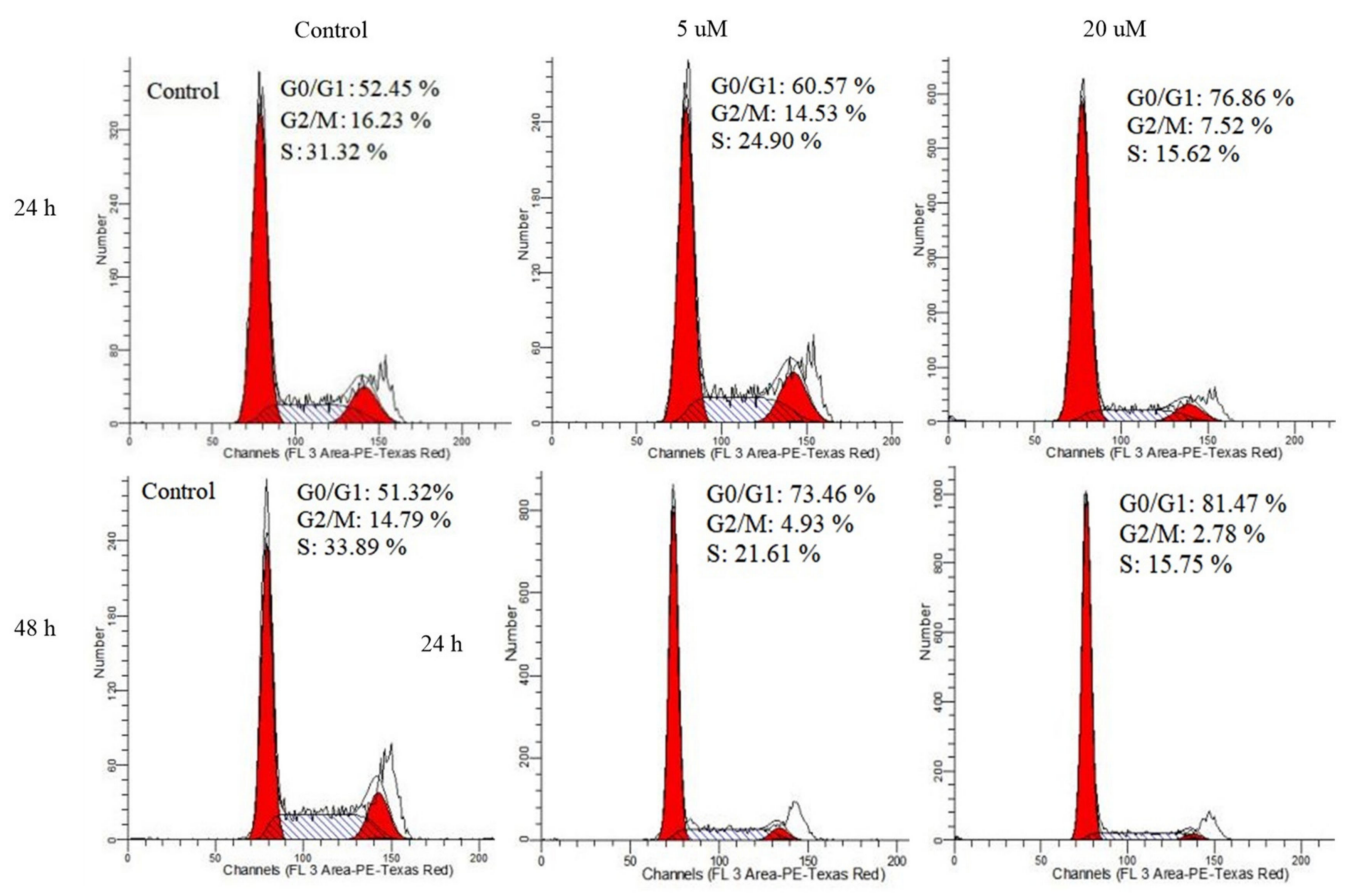
HepG2 cell cycle distributions after exposure to 5 μM and 20 μM complex **2** for 24 h and 48 h.

Cell cycle arrest plays an indispensable role in cancer cell apoptosis [49]. We used flow cytometry and PI staining to analyze HepG2 distribution at various cell stages. Figure 10 and Figure 11 show that 52.45% and 51.32% of all HepG2 cells were at G0/G1 at 24 h and 48 h, respectively. After inoculating HepG2 cells with complex **1** and complex **2** for 24 h or 48 h, the proportions of HepG2 cells in the G0/G1 phase escalated, while the proportions of HepG2 cells in G2/M and S decreased. In the control, complex **1** and complex **2** induced arrest of G0/G1 phase in HepG2 cells, and the fraction of HepG2 cells at G0/G1 was dose- and time-dependent.

### 3.6. Induced Cell Apoptosis

**Figure 12:**
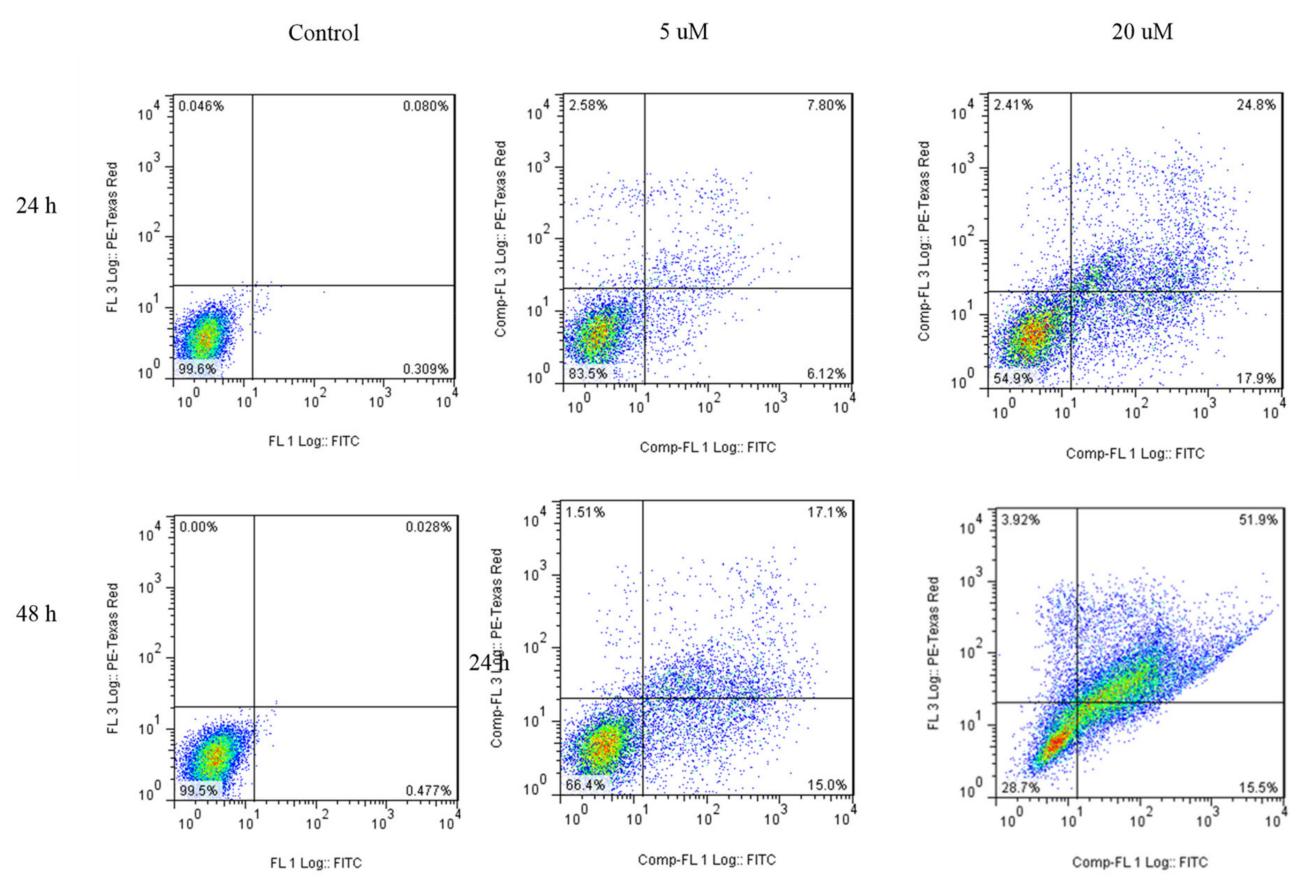
HepG2 cells inoculated with 0 μM, 5 μM, or 20 μM of complex **1** for 24 h or 48 h.

**Figure 13:**
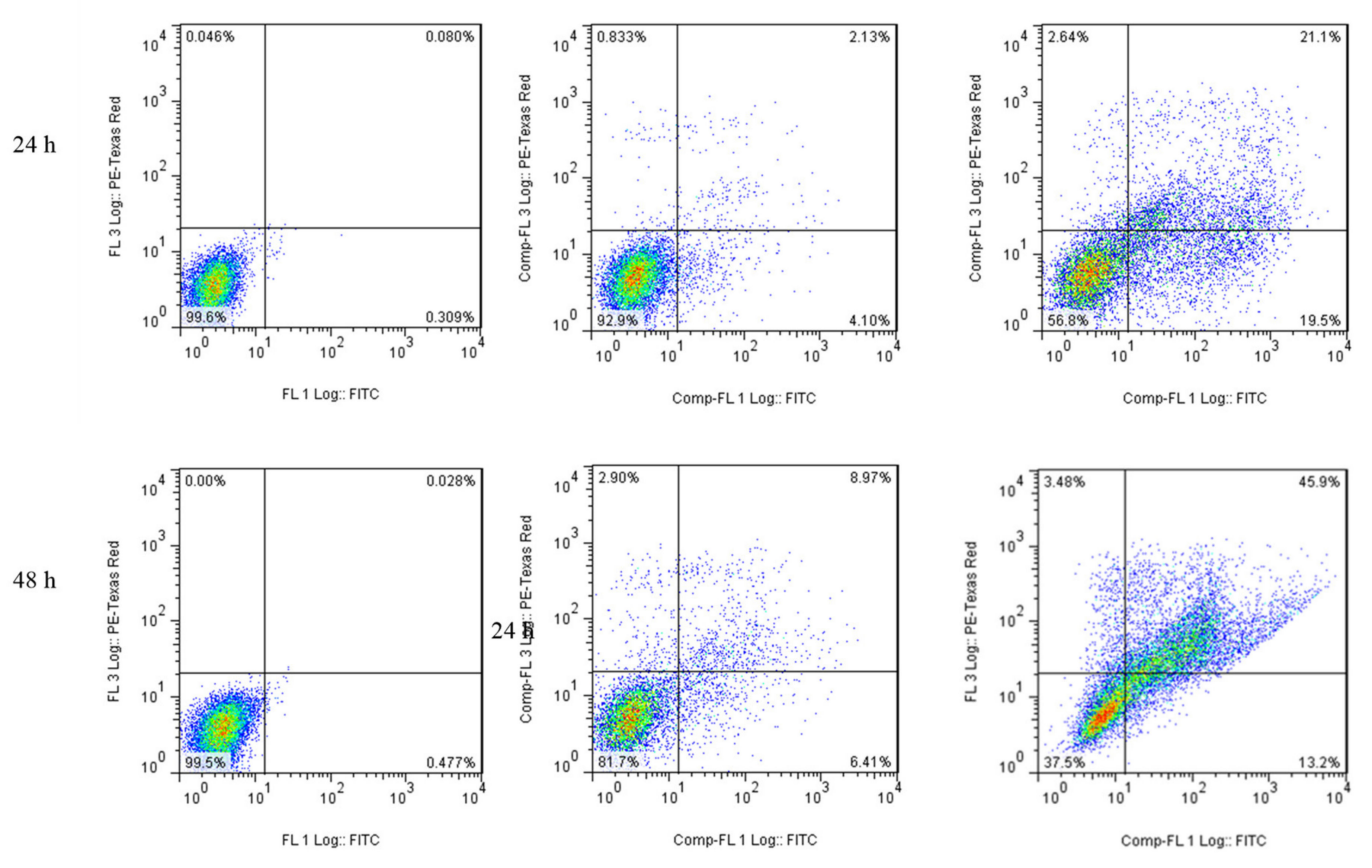
HepG2 cells inoculated with 0 μM, 5 μM, or 20 μM complex **2** for 24 h or 48 h.

Morphological apoptosis studies documented that both complexes triggered apoptosis in HepG2 cells. Apoptosis was evaluated by flow cytometry (Figure 12 and Figure 13). Untreated HepG2 cells were employed as the control. After the HepG2 cells were inoculated with 5 μM, as well as 20 μM of each complex for 24 h, the proportions of apoptotic or necrotic cells were 16.5% and 45.1% for complex **1** and 7.1% and 43.2% for complex **2**, respectively. In the control, the proportions of living and apoptotic or necrotic cells were 99.6% and 0.4%, respectively. After the HepG2 cells were inoculated with 5 μM and 20 μM of each complex for 48 h, the proportions of apoptotic or necrotic cells were 33.6% and 71.3% for complex **1** and 18.3% and 62.5% for complex **2**, respectively. In the control, the proportions of living and apoptotic cells were 99.5% and 0.5%, respectively. Relative to the control, the proportion of living cells had decreased, and the fraction of apoptotic or necrotic cells had increased in the complex treatments. Hence, the effects of the complexes on apoptosis and necrosis in HepG2 cells were dose- and time-dependent.

### 3.7. Caspase and Bcl-2 Protein Family Expression

**Figure 14:**
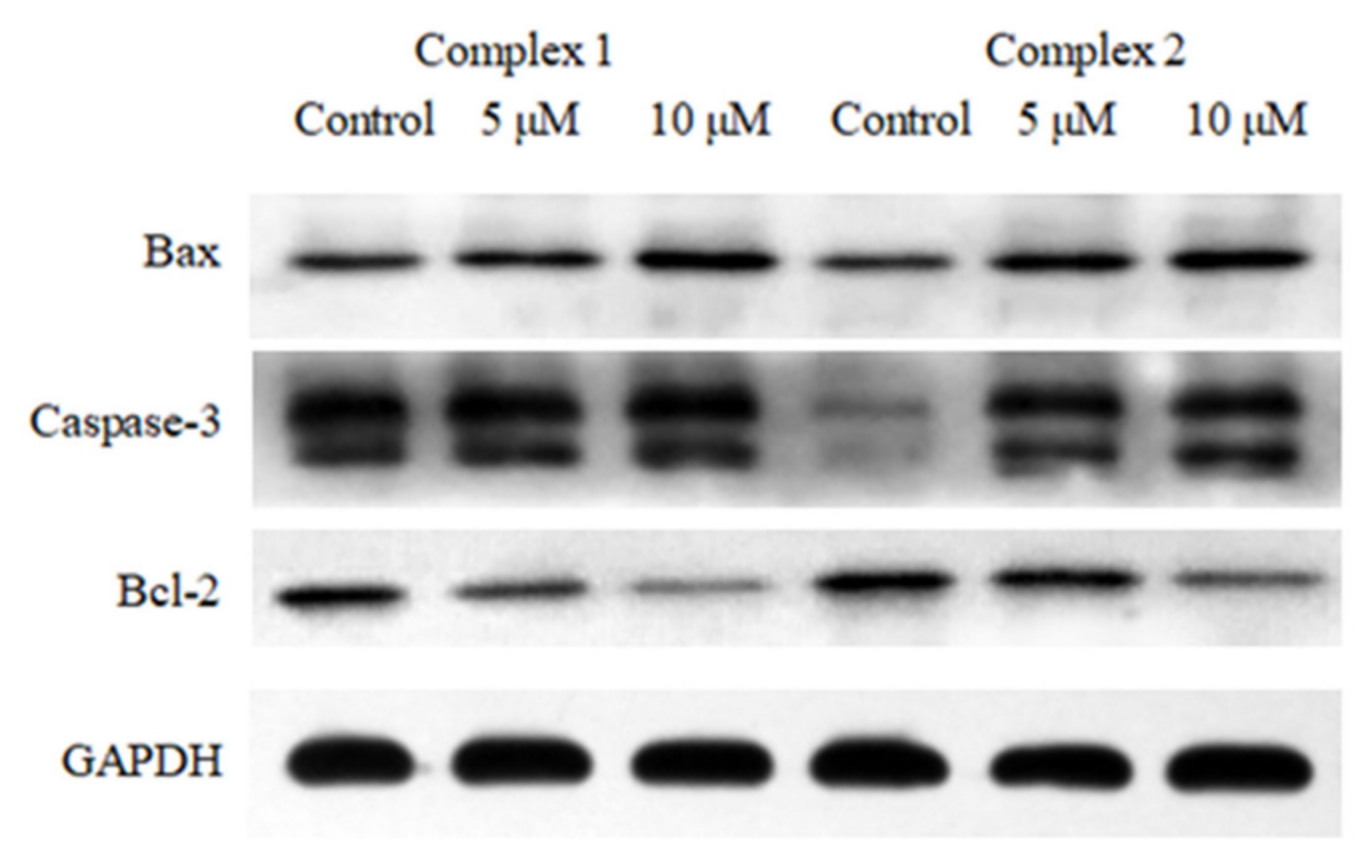
Western blot of Bax, caspase 3, and Bcl-2 in HepG2 cells inoculated with 5 μM or 10 μM complex **1** or complex **2**. GAPDH was the internal control.

Figure 14 shows that when the HepG2 cells were inoculated with 5 μM or 10 μM complex **1** or complex **2** for 48 h, caspase 3 was upregulated compared with the control. Moreover, complex **1** and complex **2** decreased the expression of the antiapoptotic protein Bcl-2 and elevated the expression of the proapoptotic protein Bax. Therefore, complex **1** and complex **2** trigger apoptosis by modulating Bcl-2 protein family expression.

## 4. Conclusions

Here, two novel copper(II) complexes were synthesized using 4-chloro-3-nitrobenzoic acid, phen, and bpy as the ligands. Single-crystal XRD showed that both complexes were monoclinic crystals with a C2/c space groups and hexacoordinated copper(II) center ions. We investigated their interactions with DNA/HSA via electronic absorption assays, fluorescence spectroscopy, and viscosity tests. Electronic absorption revealed that both copper(II) complexes intercalatively bound DNA, but complex **1** had a higher binding constant than complex **2**. Complex **1** and complex **2** statically and spontaneously quenched DNA/HSA fluorescence. In vitro cytotoxicity assays demonstrated that complex **1** was relatively more cytotoxic to HepG2, HeLa, and A549 cancer cells than complex **2** or cisplatin. This finding was concordant with the binding strength of the complexes with DNA and suggests that their anticancer activity might be associated with their potential to bind DNA. Both complex **1** and complex **2** caused HepG2 cell cycle arrest and induced G0/G1. The Annexin V-FITC/PI apoptosis assay showed that both complexes induced apoptosis or necrosis in cancer cells by modulating antiapoptotic Bcl-2 protein family expression. This study evidences the foregoing copper(II) complexes as potential anticancer drugs, which could serve in the rational development of innovative copper(II)-based antitumor agents.

## Figures and Tables

**Table 1:** Crystallographic data for complex **1** and complex **2**.

Complex	1	2
CCDC	2013056	2013057
Empirical formula	C_40_H_20_Cl_4_CuN_6_O_16_	C_38_H_20_Cl_4_CuN_6_O_16_
Formula weight	1045.96	1021.94
Temperature (K)	296(2)	296(2)
Wavelength (Å)	0.71073	0.71073
Crystal system	Monoclinic	Monoclinic
Space group	C2/c	C2/c
a (Å)	21.8874(6)	21.7978(6)
b (Å)	13.7262(4)	13.8337(4)
c (Å)	14.1778(5)	14.0676(4)
α (°)	90	90
β (°)	101.336(3)	100.120(2)
γ (°)	90	90
Volume (Å^3^)	4,176.4(2)	4,176.0(2)
Z	4	4
*Dcalcd*. (Mg·m^−3^)	1.664	1.625
*μ* (mm^−1^)	0.862	0.860
F (000)	2108	2060
Crystal size (mm)	0.31 × 0.25 × 0.14	0.24 × 0.16 × 0.14
θ (°)	1.90–25.00	1.90–25.00
Limiting indices	−25 ≤ h ≤ 26, −15 ≤ k ≤ 16, −16 ≤ l ≤ 16	−25 ≤ h ≤ 23, −16 ≤ k ≤ 15, −16 ≤ l ≤ 15
Reflections collected/unique	16,512/3692 [R_int_ = 0.0223]	14,725/3682 [R_int_ = 0.0191]
Data/restraints/parameters	3692/0/311	3682/0/301
Goodness-of-fit on F^2^	1.104	1.081
Final R indices [I > 2sigma(I)]	R_1_ = 0.0385, wR_2_ = 0.1157	R_1_ = 0.0420, wR_2_ = 0.1194
R indices (all data)	R_1_ = 0.0434, wR_2_ = 0.1240	R_1_ = 0.0484, wR_2_ = 0.1253
Largest diff. peak and hole (e/Å^3^)	0.758 and −0.618	0.669 and −0.414

**Table 2:** IC_50_ of complex 1 and complex 2 against A549, HeLa, and HepG2 at various time points.

Sample	IC_50_ (μM)			
	t (h)	A549	HeLa	HepG2
1	24	41.29 ± 1.03	28.50 ± 1.22	31.40 ± 0.63
	48	11.60 ± 0.94	9.93 ± 1.36	11.86 ± 0.78
	72	6.91 ± 0.82	8.00 ± 1.29	7.89 ± 0.87
	96	4.88 ± 0.79	5.89 ± 1.81	6.46 ± 0.87
2	24	145.01 ± 0.81	92.16 ± 0.91	58.25 ± 1.02
	48	45.03 ± 1.02	37.58 ± 0.85	27.99 ± 0.94
	72	34.59 ± 1.01	29.96 ± 0.77	23.59 ± 0.80
	96	26.08 ± 0.90	22.14 ± 0.68	17.97 ± 0.83
ncba	48	>10,000	>10,000	>1000
cisplatin	48	36.50 ± 1.76	37.37 ± 2.01	43.81 ± 1.62

## Data Availability

CCDC 2013056 and 2013057 contain the supplementary crystallographic data for this paper. These data can be obtained free of charge via www.ccdc.cam.ac.uk/data_request/cif, or by emailing data_request@ccdc.cam.ac.uk, or by contacting The Cambridge Crystallographic Data Centre.

## Supplementary Materials

The following are available online. Figure S1: ^1^H NMRspectrum of cnba, Figure S2: IR spectrum of the complex 1, Figure S3: IR spectrum of the complex 2, Table S1: Bond lengths and bond angles parameters for complexes 1 and 2.

## Sample Availability

Samples of Complex **1** and complex **2** are available from the authors.

## Abbreviations

CHNS	carbon hydrogen nitrogen sulfur
EB	ethidium bromide
ncba	4-chloro-3-nitrobenzoic acid
phen	1,10-phenanthroline
bpy	2,2′-bipyridine
CCK-8	cell counting kit 8
CT-DNA	calf thymus DNA
HeLa	human cervical carcinoma
HepG2	human hepatocellular carcinoma
HSA	human serum albumin
A549	human lung carcinoma
Bax	Bcl-2-associated X protein
BSA	bovine serum albumin
Bcl-2	B cell lymphoma 2
PBS	phosphate-buffered saline
PI	propidium iodide
