# Supplementary material for: Synthesis, Characterization, DNA/HSA Interactions, and Anticancer Activity of Two Novel Copper(II) Complexes with 4-Chloro-3-Nitrobenzoic Acid Ligand
Source: Molecules. 2021 Jul 1;26(13):4028. doi: 10.3390/molecules26134028 (PMC8271622; doi:10.3390/molecules26134028)

checkCIF/PLATON report

You have not supplied any structure factors. As a result the full set of tests cannot be run.

THIS REPORT IS FOR GUIDANCE ONLY. IF USED AS PART OF A REVIEW PROCEDURE FOR PUBLICATION, IT SHOULD NOT REPLACE THE EXPERTISE OF AN EXPERIENCED CRYSTALLOGRAPHIC REFEREE.

No syntax errors found. CIF dictionary Interpreting this report

Datablock: nzf-35-300k

Bond precision: C-C = 0.0046 A Wavelength=0.71073

Cell:	a=21.7978(6)	b=13.8337(4)	c=14.0676(4)
	alpha=90	beta=100.120(2)	gamma=90
Temperature:	296 K		

	Calculated	Reported
Volume	4176.0(2)	4176.0(2)
Space group	C 2/c	C 1 2/c 1
Hall group	-C 2yc	-C 2yc
Moiety formula	C38 H20 Cl4 Cu N6 O16	C24 H14 Cl2 Cu N4 O8, 2(C7 H3 Cl N O4)
Sum formula	C38 H20 Cl4 Cu N6 O16	C38 H20 Cl4 Cu N6 O16
Mr	1021.95	1021.94
Dx,g cm-3	1.625	1.625
Z	4	4
Mu (mm-1)	0.860	0.860
F000	2060.0	2060.0
F000’	2064.66	
h,k,lmax	25,16,16	25,16,16
Nref	3683	3682
Tmin,Tmax	0.848,0.887	0.835,1.000
Tmin’	0.814	

Correction method= # Reported T Limits: Tmin=0.835 Tmax=1.000
AbsCorr = MULTI-SCAN

Data completeness= 1.000 Theta(max)= 25.000

R(reflections)= 0.0420(3190) wR2(reflections)= 0.1253(3682)

S = 1.081 Npar= 301

The following ALERTS were generated. Each ALERT has the format
test-name_ALERT_alert-type_alert-level.
Click on the hyperlinks for more details of the test.

Alert level C

```
PLAT193_ALERT_1_C Cell and Diffraction Temperatures Differ by ....          3 Degree
PLAT220_ALERT_2_C NonSolvent   Resd 1  O   Ueq(max)/Ueq(min) Range        3.4 Ratio
PLAT241_ALERT_2_C High   'MainMol' Ueq as Compared to Neighbors of         O6 Check
PLAT242_ALERT_2_C Low    'MainMol' Ueq as Compared to Neighbors of         N2 Check
PLAT309_ALERT_2_C Single Bonded Oxygen (C-O > 1.3 Ang) ..........         O5 Check
```

Alert level G

```
PLAT005_ALERT_5_G No Embedded Refinement Details Found  in the CIF     Please Do !
PLAT042_ALERT_1_G Calc. and Reported MoietyFormula Strings  Differ     Please Check
PLAT083_ALERT_2_G SHELXL Second Parameter in WGHT  Unusually Large       6.39 Why ?
PLAT301_ALERT_3_G Main Residue  Disorder ..............(Resd  1  )         6% Note
PLAT395_ALERT_2_G Deviating  X-O-Y    Angle From 120 for O3A             52.4 Degree
PLAT395_ALERT_2_G Deviating  X-O-Y    Angle From 120 for O4A             48.5 Degree
PLAT432_ALERT_2_G Short Inter X...Y Contact  O3A      ..C11              3.01 Ang.
                                  1/2+x,3/2-y,1/2+z  =      8_566 Check
PLAT779_ALERT_4_G Suspect or Irrelevant (Bond) Angle(s) in CIF . #         14 Check
              O3A  -N2   -O3B     1.555   1.555   1.555             39.80 Deg.
PLAT794_ALERT_5_G Tentative Bond Valency for Cu1       (II)      .       2.23 Info
```

```
   0 ALERT level A = Most likely a serious problem - resolve or explain
   0 ALERT level B = A potentially serious problem, consider carefully
   5 ALERT level C = Check. Ensure it is not caused by an omission or oversight
   9 ALERT level G = General information/check it is not something unexpected

   2 ALERT type 1 CIF construction/syntax error, inconsistent or missing data
   8 ALERT type 2 Indicator that the structure model may be wrong or deficient
   1 ALERT type 3 Indicator that the structure quality may be low
   1 ALERT type 4 Improvement, methodology, query or suggestion
   2 ALERT type 5 Informative message, check
```

Validation response form

Please find below a validation response form (VRF) that can be filled in and pasted into your CIF.

```
# start Validation Reply Form
_vrf_PLAT193_nzf-35-300k
;
PROBLEM: Cell and Diffraction Temperatures Differ by ....          3 Degree
RESPONSE: ...
;
_vrf_PLAT220_nzf-35-300k
;
PROBLEM: NonSolvent   Resd 1  O   Ueq(max)/Ueq(min) Range        3.4 Ratio
RESPONSE: ...
;
_vrf_PLAT241_nzf-35-300k
;
PROBLEM: High   'MainMol' Ueq as Compared to Neighbors of         O6 Check
RESPONSE: ...
;
_vrf_PLAT242_nzf-35-300k
;
```

```
PROBLEM: Low    'MainMol' Ueq as Compared to Neighbors of         N2 Check
RESPONSE: ...
;
_vrf_PLAT309_nzf-35-300k
;
PROBLEM: Single Bonded Oxygen (C-O > 1.3 Ang) ...........         O5 Check
RESPONSE: ...
;
# end Validation Reply Form
```

It is advisable to attempt to resolve as many as possible of the alerts in all categories. Often the minor alerts point to easily fixed oversights, errors and omissions in your CIF or refinement strategy, so attention to these fine details can be worthwhile. In order to resolve some of the more serious problems it may be necessary to carry out additional measurements or structure refinements. However, the purpose of your study may justify the reported deviations and the more serious of these should normally be commented upon in the discussion or experimental section of a paper or in the "special_details" fields of the CIF. checkCIF was carefully designed to identify outliers and unusual parameters, but every test has its limitations and alerts that are not important in a particular case may appear. Conversely, the absence of alerts does not guarantee there are no aspects of the results needing attention. It is up to the individual to critically assess their own results and, if necessary, seek expert advice.

Publication of your CIF in IUCr journals

A basic structural check has been run on your CIF. These basic checks will be run on all CIFs submitted for publication in IUCr journals (*Acta Crystallographica*, *Journal of Applied Crystallography*, *Journal of Synchrotron Radiation*); however, if you intend to submit to *Acta Crystallographica Section C* or *E* or *IUCrData*, you should make sure that full publication checks are run on the final version of your CIF prior to submission.

Publication of your CIF in other journals

Please refer to the *Notes for Authors* of the relevant journal for any special instructions relating to CIF submission.

PLATON version of 05/12/2020; check.def file version of 05/12/2020

Datablock nzf-35-300k - ellipsoid plot

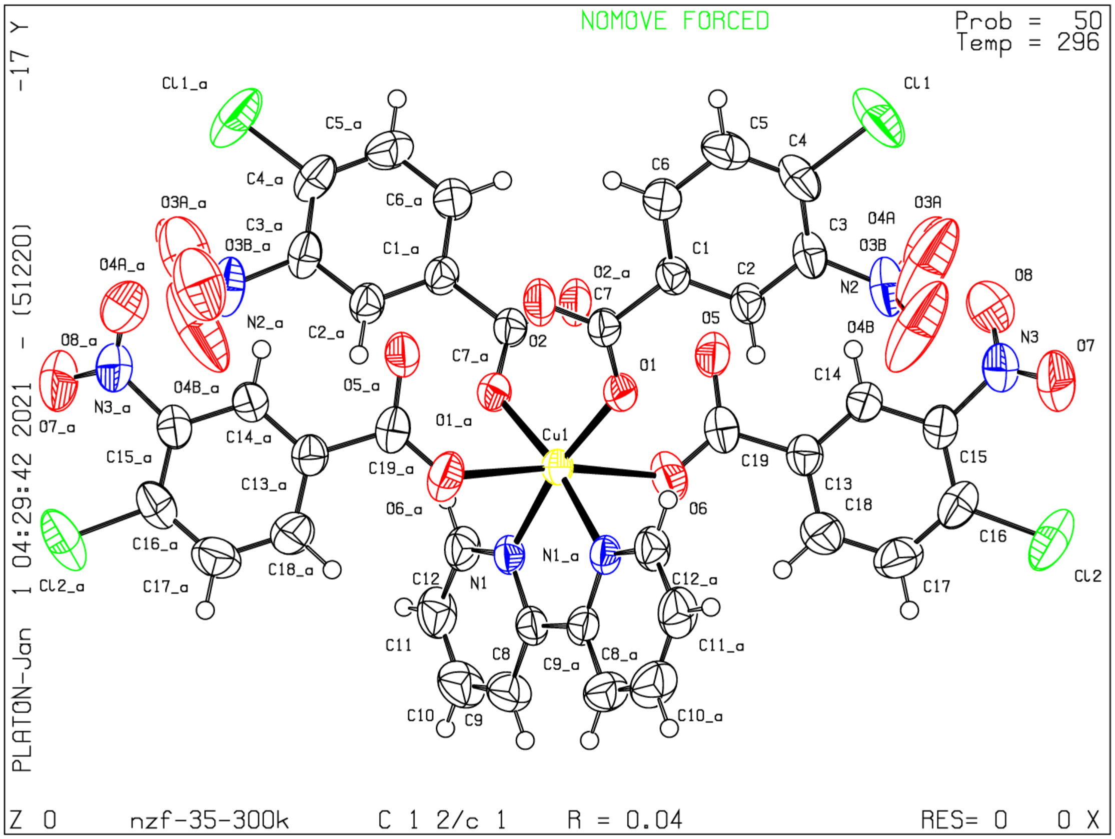

Supplement: Supplementary file 1 [file molecules-26-04028-s001.zip › Crystallographic Data/2checkcif.pdf]
