# Supplementary material for: Synthesis, Characterization, DNA/HSA Interactions, and Anticancer Activity of Two Novel Copper(II) Complexes with 4-Chloro-3-Nitrobenzoic Acid Ligand
Source: Molecules. 2021 Jul 1;26(13):4028. doi: 10.3390/molecules26134028 (PMC8271622; doi:10.3390/molecules26134028)
Supplement: Supplementary file 1 [file molecules-26-04028-s001.zip › Supporting Information.pdf]

Supporting Information

Article

Synthesis, characterization, DNA/HSA interactions, and anticancer activity of two novel Copper(II) complexes with 4-chloro-3-nitrobenzoic acid ligand

Zhen-Fang Zeng[1,*], Qiu-Ping Huang[1], Jie-Hui Cai[1], Guang-Jin Zheng[1], Qiu-Chan Huang[1], Zi-Lu Liu[1], Zi-Lu Chen[2,*], You-Huan Wei[1,*]

1 School of Chemical and Biological Engineering, Guangxi Normal University for Nationalities, 23 Fozi Road, Chongzuo 532200, PR China
2 State Key Laboratory for the Chemistry and Molecular Engineering of Medicinal Resources, School of Chemistry and Pharmacy, Guangxi Normal University, 15 Yucai Road, Guilin 541004, PR China
* Corresponding author: zengzhenfang@gxnun.edu.cn, zlchen@mailbox.gxnu.edu.cn, weiyouhuan@gxnun.edu.cn.
Telephone/Fax: 86-771-787-0799

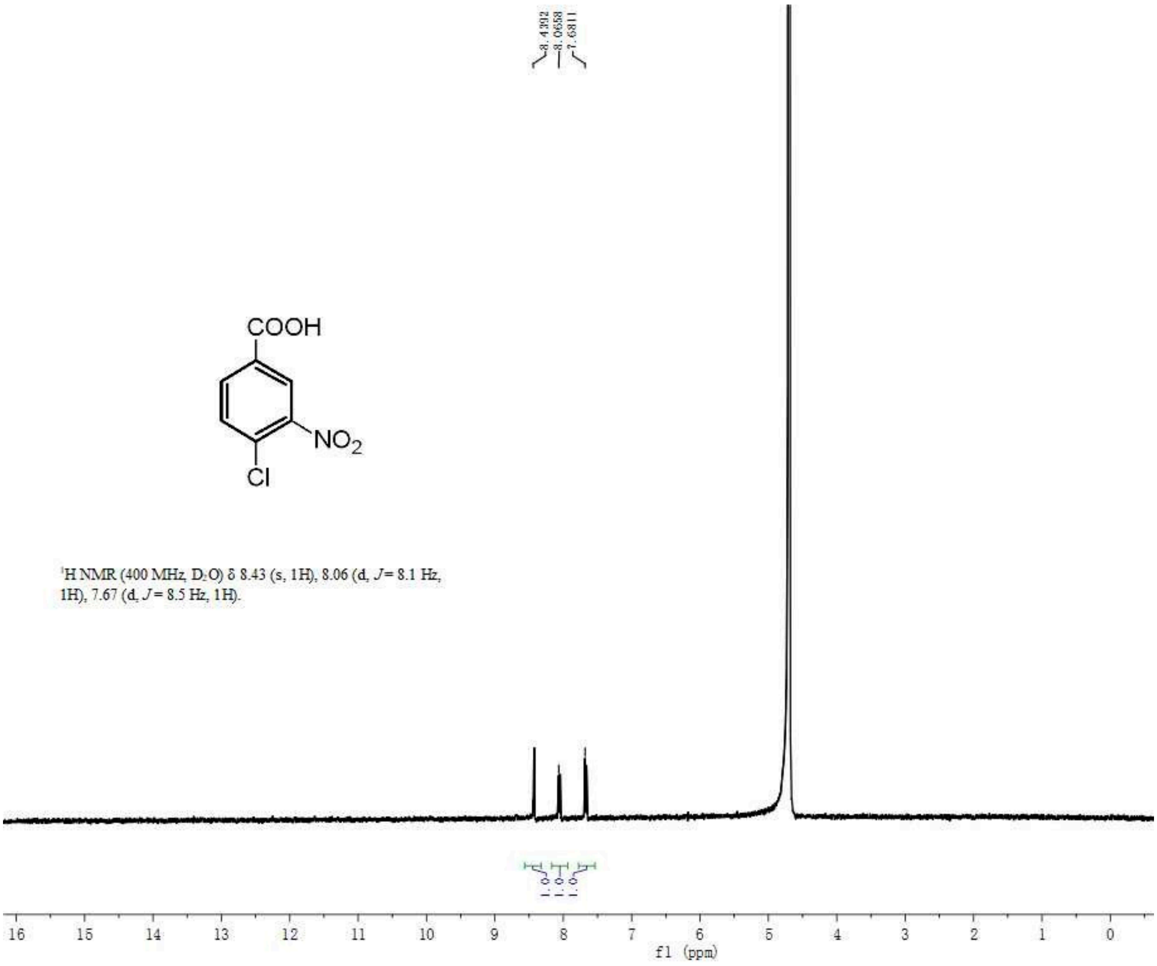

Figure S1. ^{1}H NMRspectrum of cnba.

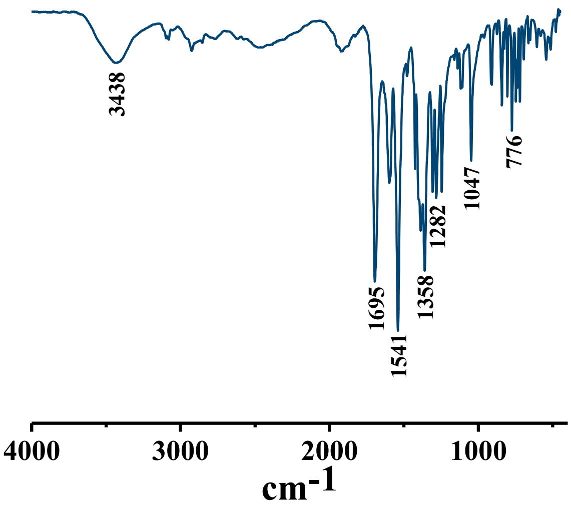

Figure S2. IR spectrum of the complex 1.

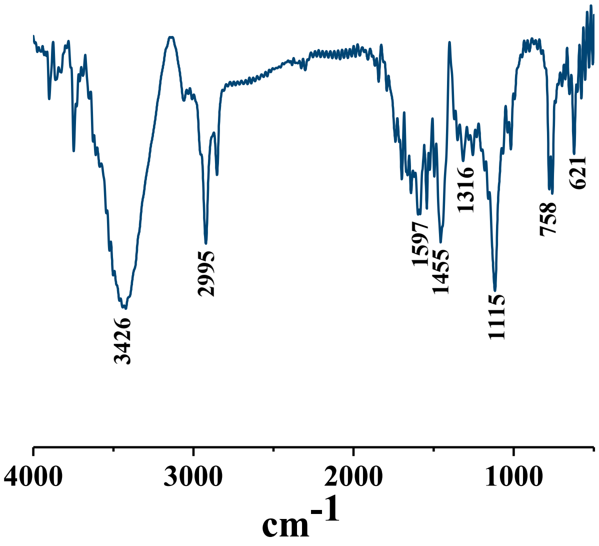

Figure S3. IR spectrum of the complex 2.

Table S1. Bond lengths and bond angles parameters for complexes **1** and **2**.

Bond length (Å)							
Complex **1**				Complex **2**			
Cu(1)-O(1)	1.9354(17)	Cu(1)-N(1)#1	2.010(2)	Cu(1)-O(1)	1.9380(18)	Cu(1)-N(1)	1.999(2)
Cu(1)-O(1)#1	1.9354(17)	Cu(1)-N(1)	2.010(2)	Cu(1)-O(1)#1	1.9380(18)	Cu(1)-N(1)#1	1.999(2)

Bond angle (°)			
Complex **1**		Complex **2**	
O(1)-Cu(1)-O(1)#1	97.03(11)	O(1)-Cu(1)-O(1)#1	96.54(12)
O(1)#1-Cu(1)-N(1)	91.11(8)	O(1)#1-Cu(1)-N(1)#1	169.97(8)
O(1)#1-Cu(1)-N(1)#1	168.87(7)	O(1)-Cu(1)-N(1)#1	91.47(9)
O(1)-Cu(1)-N(1)	168.87(7)	O(1)-Cu(1)-N(1)	169.97(8)
O(1)-Cu(1)-N(1)#1	91.11(8)	O(1)#1-Cu(1)-N(1)	91.47(9)
N(1)-Cu(1)-N(1)#1	81.90(11)	N(1)-Cu(1)-N(1)#1	81.25(13)
C(7)-O(1)-Cu(1)	131.60(16)	C(7)-O(1)-Cu(1)	131.76(18)
C(15)-N(1)-Cu(1)	112.53(15)	C(8)-N(1)-Cu(1)	114.68(17)
C(20)-N(1)-Cu(1)	128.95(17)	C(12)-N(1)-Cu(1)	126.0(2)
